# New perspectives of cobalt tris(bipyridine) system: anti-cancer effect and its collateral sensitivity towards multidrug-resistant (MDR) cancers

**DOI:** 10.18632/oncotarget.18991

**Published:** 2017-07-05

**Authors:** Betty Yuen Kwan Law, Yuan Qing Qu, Simon Wing Fai Mok, Hauwei Liu, Wu Zeng, Yu Han, Flora Gordillo-Martinez, Wai-Kit Chan, Keith Man-Chung Wong, Vincent Kam Wai Wong

**Affiliations:** ^1^ State Key Laboratory of Quality Research in Chinese Medicine, Macau University of Science and Technology, Macau, P.R. China; ^2^ Department of Chemistry, South University of Science and Technology of China, Tangchang Boulevard, Nanshan District, Shenzhen, P.R. China

**Keywords:** cobalt complexes, collateral sensitivity, autophagy, anti-cancer, drug-resistant cancer

## Abstract

Platinating compounds including cisplatin, carboplatin, and oxaliplatin are common chemotherapeutic agents, however, patients developed resistance to these clinical agents after initial therapeutic treatments. Therefore, different approaches have been applied to identify novel therapeutic agents, molecular mechanisms, and targets for overcoming drug resistance. In this study, we have identified a panel of cobalt complexes that were able to specifically induce collateral sensitivity in taxol-resistant and p53-deficient cancer cells. Consistently, our reported anti-cancer functions of cobalt complexes 1–6 towards multidrug-resistant cancers have suggested the protective and non-toxic properties of cobalt metal-ions based compounds in anti-cancer therapies. As demonstrated in xenograft mouse model, our results also confirmed the identified cobalt complex 2 was able to suppress tumor growth *in vivo*. The anti-cancer effect of the cobalt complex 2 was further demonstrated to be exerted via the induction of autophagy, cell cycle arrest, and inhibition of cell invasion and P-glycoprotein (P-gp) activity. These data have provided alternative metal ion compounds for targeting drug resistance cancers in chemotherapies.

## INTRODUCTION

Transition metal ions are essential for the proper functions of organisms; examples including copper, iron, and manganese ions work with proteins and enzymes for multiple biological processes such as electron transfer and catalysis. As metals are involved in redox activity, coordination, and reactivity towards organic substrates in organisms, and are tightly regulated under normal conditions, aberrant metal ion concentrations are associated with pathogenesis of diseases, in particular of cancers. For instance, enriched copper ions found in cancer tissues are suggested to promote the angiogenesis processes in tumors. The use of copper ion-binding ligands is therefore anticipated to provide a novel anti-cancer therapy [1].

In fact, metal-containing compounds have been used to treat a wide range of diseases. For example, cisplatin (cis-[Pt^II^(NH_3_)_2_Cl_2_]) can bind to the purine bases of DNA, thereby led to DNA damage resulting in apoptosis in cancer cells. However, the clinical use of cisplatin is limited due to severe side effects such as dose-dependent toxicity, allergy, problems on kidney and immunity, gastrointestinal disorders, hemorrhage and loss of hearing. Acquired resistance to cisplatin is caused by an increased efflux or detoxification of the drug, increased rate of DNA repair, as well as the less susceptible of cancer cells in response to drug-induced cell death [1]. Other platinum-containing anti-cancer analogs such as carboplatin and oxaliplatin are therefore used as alternative of cisplatin [2]. Other transition metal complexes including zinc(II), copper(II), gold(III), copper chelating agents, and non-platinum metal complexes such as ruthenium-containing compounds were therefore studied for their potential as anti-cancer agents [1, 3–7].

The exploration and exploitation of other non-platinum anti-cancer drugs have been receiving considerable attention. In view of the fact that soluble cobalt salts can interfere with cell division adversely, and bind to nucleic acids inside the cell nucleus, one may postulate that cobalt complexes could work as anti-cancer agent like platinum-containing analogs. However, it is also weakly mutagenic and induces metastasis in animal models [8]. There have been some examples of cobalt(III) complexes with equatorial tetradentate Schiff base ligand as potent inhibitors of a wide array of zinc-dependent proteins [9–12]. However, the related use of cobalt polypyridine complexes in biological application or specifically for the development of anti-cancer drug was relatively unexplored [13, 14]. On the other hand, cobalt(II)/(III) complexes with polypyridine ligands have recently been developed as redox mediators in dye-sensitized solar cells (DSCs) [15–19]. The cobalt tris(bipyridine) complexes, [Co(N-N)_3_]^2+/3+^ (N-N = bipyridine), are a class of promising candidates for replacement of I^−^/I_3_^−^ couple as iodine-free electrolytes in such SDCs. Unlike the cisplatin with coordination unsaturated square-planar geometry, studies of the octahedral transition metal complexes on the anti-cancer properties are relatively rare. Herein, we report new perspectives for the application of a series of cobalt tris(bipyridine) complexes with different oxidation states and substituents on the bipyridine ligand. To the best of our knowledge, this is the first research work which reported promising anti-cancer effect and, more importantly, their collateral sensitivity that target the multidrug-resistant cancers instead the drug-sensitive parental cells from which they were derived.

## RESULTS

### Synthesis and characterizations

**Figure 1:**
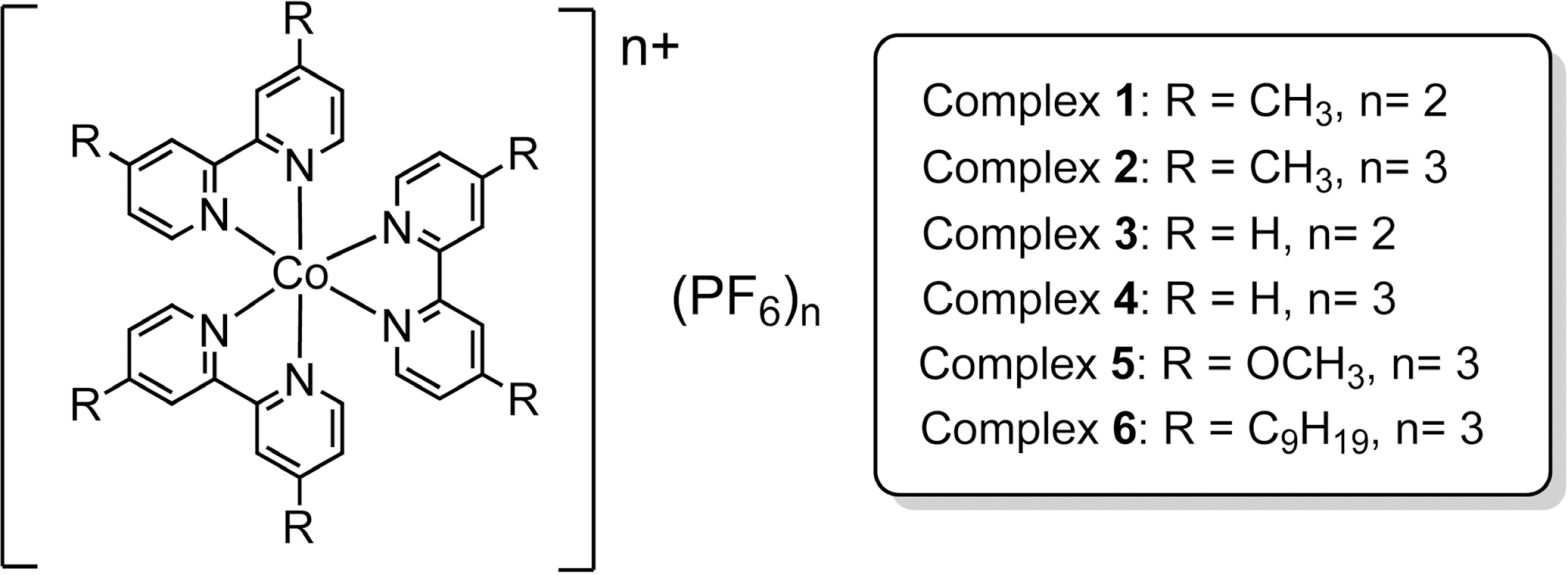
Molecular structures of cobalt complexes 1–6

Six cobalt tris(bipyridine) complexes have been selected in this work (Figure 1). The oxidation state of +2 and +3 represent the high-spin and low-spin electronic configurations, respectively, while the CH_3_, OCH_3_ and C_9_H_19_ substituents on the bipyridine ligand determine the electronic effect, size of the coordination sphere as well as the lipophilicity. All of them are in racemic form without the purification of their optical isomers, i.e Δ and Λ forms. According to the modification of reported procedures [18], complexes 1–6 have been prepared and characterized. The water soluble form of complex 2, 2W, in chloride salt was prepared by metathesis reaction in acetone, by using lithium chloride.

### Potent anti-cancer effect towards a panel of cancer cells

Metal ion containing compounds have been demonstrated to exhibit potent anti-tumor effect on various types of cancers [1]. For instance, platinum ion containing cisplatin (cis-[Pt^II^(NH_3_)_2_Cl_2_]) is the well-known FDA approved anti-cancer agent, which has been found to induce apoptosis via Akt, PKC or MAPKs signaling pathways [20] and has long been used to treat colon, ovarian and lung cancers.

Nevertheless, the anti-tumor efficacy of cisplatin is often limited in multidrug-resistant or p53-deficient cancers [21]. Therefore, discovery of other metal ion containing compounds with specific potency on multidrug-resistant cancers are desired. The present cobalt ion containing complexes 1–6 were found to exhibit potent cytotoxic effect towards a panel of cancer and normal cells, including HeLa (cervical cancer), MCF-7 (breast cancer), H1299 (lung cancer), H1975 (EGFR mutant lung cancer), A549 (EGFR wild-type lung cancer), LLC-1 (mouse lung cancer), HepG2 (liver cancer) and Hep3B (liver cancer) cells, and normal human LO2 hepatocytes. As shown in Table 1 and Supplementary Figures 1–3, complexes 1–6 displayed considerable cytotoxicity against cancer cells from different origins. Among the six cobalt complexes 1, 2 and 5 demonstrated the highest anti-cancer potency with mean IC_50_ values ranged from 2.79 – 18.6 μM, the lower the IC_50_ values, the higher the anti-cancer potency. By contrast, the cytotoxicity of complexes 1 and 2 was relatively lower in LO2 normal human hepatocytes (IC_50_: 13.5 and 13.2 μM, respectively). Beside, complexes 3 and 4 showed moderate anti-cancer potency with mean IC_50_ values ranged from 6.92 – 37.2 μM, whereas complex 6 possessed the lowest anti-cancer potency with mean IC_50_ values ranged from 10.4 – 82.8 μM. The complexes in different oxidation states (or in different spin states) with the same substituents on the bipyridine ligand (1 and 2; 3 and 4) showed similar cytotoxcity effect in the cancer cells. Although the cobalt(II) and cobalt(III) complexes are considered as inert d^5^ and lable d^6^ species, respectively, for bioreductive activation as redox-activated prodrugs through ligand modification [9], there is no direct relationship in the nature of spin state on the cytotoxcity effect. Complex 5 having OCH_3_ group generally exhibited higher anti-cancer potency to 4 with un-substituted bipyridine ligand whereas less effective to 2 with CH_3_ group. On the other hand, the larger coordination sphere size and higher lipophilicity may account for the high IC_50_ values of complex 6. By scrutinizing their anti-cancer potencies, it is noteworthy that complex 2 was found to exhibit specific cytotoxic effects towards cancer cells by demonstrating the lowest mean IC_50_ values in various types of cancer cell lines.

**Table 1:** Mean IC_50_ values [μM] of cobalt complexes 1–6 in various cell lines

	1	2	3	4	5	6
**HeLa**	10.1	11.9	24.2	35.9	12.8	21.3
**MCF-7**	15.4	6.03	15.8	10.1	10.8	37.4
**H1299**	3.43	2.84	9.4	14.9	8.71	26.7
**H1975**	15.4	8.84	17.0	21.0	9.31	24.2
**A549**	9.45	7.86	26.3	37.2	18.6	82.8
**LLC-1**	2.79	3.39	17.2	29.2	8.71	10.4
**HepG2**	7.39	5.44	13.3	22.7	15.4	26.0
**Hep 3B**	4.06	3.7	6.92	13.8	5.33	54.1
**LO2**	13.5	13.2	33.6	43.7	20.6	40.2

### Collateral sensitivity in taxol-resistant and p53-deficient cancer cells

Together with genetically defect-mediated drug-resistance or apoptosis-resistance, multidrug resistance (MDR) of neoplastic tissue represents a real obstacle in effective treatment of disseminated cancers [22, 23]. In the current study, taxol-resistant, cisplatin-resistant and p53-deficient cancer cells from different origins, including breast cancer MCF-7, colon cancer HCT-8 and lung cancer A549 taxol-sensitive/resistant cells, ovarian cancer A2780, gastric cancer SGC-7901, OV2008 and C13 cisplatin-sensitive/resistant cells, and p53-wild-type/deficient colon cancer cells, HCT116 were adopted to evaluate the therapeutic potential of complexes 1–6 towards multidrug-resistant cancers. As shown in Table 2 and Supplementary Figures 4–6, taxol treatment (positive control) of the taxol-resistant phenotype of MCF-7, HCT-8, and A549 demonstrated higher mean IC_50_ values when compared to the sensitive counterparts as expected with resistant factor (IC_50_ values of taxol-resistant cells over the IC_50_ values of taxol-sensitive cells) up to 42.4. In contrast, nearly all cobalt complexes in this study displayed lower mean IC_50_ values in taxol-resistant cancers when compared to their taxol-sensitive cells in MCF-7, HCT-8, and A549 cancer, with resistant factor below 0.8, suggesting that these cobalt complexes exhibit collateral sensitivity towards the taxol-resistant cancers. Compared with the cytotoxicity effect to the drug-sensitive cancer, complexes 1, 2 and 5 demonstrated the most potent cytotoxic effect towards these taxol-resistant cancers. In addition, cisplatin-sensitive and -resistant pair of cancer cells including A2780 ovarian cancer, SGC-7901 gastric cancer, OV2008 and C13 cervical cancer were further adopted to examine the collateral sensitivity of selected cobalt complexes (Table 3 and Supplementary Figures 7–9). In the positive control, our cisplatin-resistant cellular models were treated with cisplatin and displayed higher mean IC_50_ values than the sensitive counterparts with resistant factor up to 13.25. It is interesting to note that nearly all six cobalt complexes exhibited similar cytotoxic effect towards both cisplatin-sensitive and -resistant cancers, with resistant factor close to 1, strongly suggesting the cross-sensitive effect on cisplatin-resistant cancers by these cobalt complexes. These findings indicate that although cobalt complexes didn't show increased selective (collateral) sensitivity in cisplatin-resistant cancer cells, these cobalt complexes still exhibit good potency in both cisplatin-sensitive and -resistant cancer, especially in complex 1 and 2. This result suggests that the cobalt complexes may be a good candidate to replace cisplatin in treatment of cisplatin-resistant cancer cells.

**Table 2:** Collateral sensitivity of cobalt complexes 1–6 in taxol-resistant cancer cells

	Cancer Types	IC_50_ value, μM	IC_50_ value, μM	Resistant Factor
		Taxol-sensitive cancer	Taxol-resistant cancer	
	MCF-7	12.2	55	4.51
**Taxol**	HCT-8	12.6	>200	>15.9
	A549	1.56	66.1	42.4
1	MCF-7	4.62	2.51	0.54
	HCT-8	3.13	1.16	0.37
	A549	9.59	2.37	0.25
2	MCF-7	4	2.71	0.68
	HCT-8	3.39	1.22	0.36
	A549	11.5	2.85	0.25
3	MCF-7	13.4	16.8	1.25
	HCT-8	11.4	7.78	0.68
	A549	29.9	7.85	0.26
4	MCF-7	8.02	5.01	0.62
	HCT-8	18.5	10.2	0.55
	A549	51.7	11.1	0.21
5	MCF-7	9.45	6.28	0.66
	HCT-8	4.3	1.04	0.24
	A549	15	2.85	0.19
6	MCF-7	28.5	11.1	0.39
	HCT-8	56.4	41.1	0.73
	A549	54.3	2.6	0.05

**Table 3:** Collateral sensitivity of cobalt complexes 1–6 in cisplatin-resistant cancer cells

	Cancer Types	IC_50_ value, μM	IC_50_ value, μM	Resistant Factor
		cisplatin-sensitive cancer	cisplatin-resistant cancer	
	A2780	15.1	> 200	> 13.25
**Cisplatin**	SGC-7901	3.92	43	10.97
	OV2008	18.2	–	> 11.0
	C13	–	> 200	
1	A2780	11.9	11.2	0.94
	SGC-7901	11.7	17.6	1.50
	OV2008	7.06	–	2.21
	C13	–	15.6	
2	A2780	12.3	10.7	0.87
	SGC-7901	11.1	12.6	1.14
	OV2008	7.62	–	1.0
	C13	–	7.59	
3	A2780	28.8	31.6	1.10
	SGC-7901	29.9	29.9	1.00
	OV2008	18.9	–	0.79
	C13	–	14.9	
4	A2780	55	54.1	0.98
	SGC-7901	41.1	41.7	1.01
	OV2008	42.5	–	0.88
	C13	–	37.4	
5	A2780	24	27	1.13
	SGC-7901	21.6	20.7	0.96
	OV2008	14	–	1.45
	C13	–	20.3	
6	A2780	30.2	29.1	0.96
	SGC-7901	36.3	35.9	0.99
	OV2008	49.4	–	1.87
	C13	–	92.6	

Apart from the drug-mediated resistant phenotype, genetic defect-mediated drug resistance also causes a significant burden in effective treatment of cancer [24]. For instance, p53 tumor suppressor gene is commonly mutated in 50% of cancer patients [25]. Genomic analysis of various types of human tumor cell lines demonstrated a positive correlation between p53 mutations with chemo-resistance and apoptosis-resistance [25]. We therefore further assessed the collateral sensitivity of complexes 1–6 in a pair of p53-deficient isogenic colon cancer cells. Only complexes 1, 2 and 5 were found to display collateral sensitivity in p53-deficient colon cancer with resistant factor ranged from 0.52 to 0.59 (Table 4 and Supplementary Figure 10), whereas 1 and 2 demonstrated the most potent and lowest IC_50_ values in p53-deficient colon cancer. Collectively, 1 and 2 complexes are effective in treatment of taxol-resistant and p53-deficient apoptosis-resistant cancer. In addition, these two complexes also demonstrate good potency in cisplatin-resistant cancer as well. Since p53 mutation contributes to drug-resistant or apoptosis-resistant phenotypes [26], and overexpression of P-glycoprotein (P-gp) or drug efflux pump is commonly found in taxol-resistant cancer cells [27]. As the cobalt complexes 1 and 2 induced a stronger cytotoxic effect towards cancer cells with hampered p53 expression, these complexes would not be the substrate of P-gp, and could enter and kill the MDR cancer cells effectively.

**Table 4:** Collateral sensitivity of cobalt complexes 1–6 in p53 mutant colon cancer cells

Cobalt Complex	IC_50_ value, μM	IC_50_ value, μM	Resistant Factor
	HCT116 p53^+/+^ colon cancer	HCT116 p53^-/-^ colon cancer	
**1**	17.1	8.89	0.52
**2**	15.4	9.07	0.59
**3**	34.3	29.1	0.85
**4**	45.1	45	1.00
**5**	77.8	41.3	0.53
**6**	36.5	34.1	0.93

### Cancer cells growth inhibition via autophagy induction, cell cycle arrest and cell invasion inhibition

**Figure 2:**
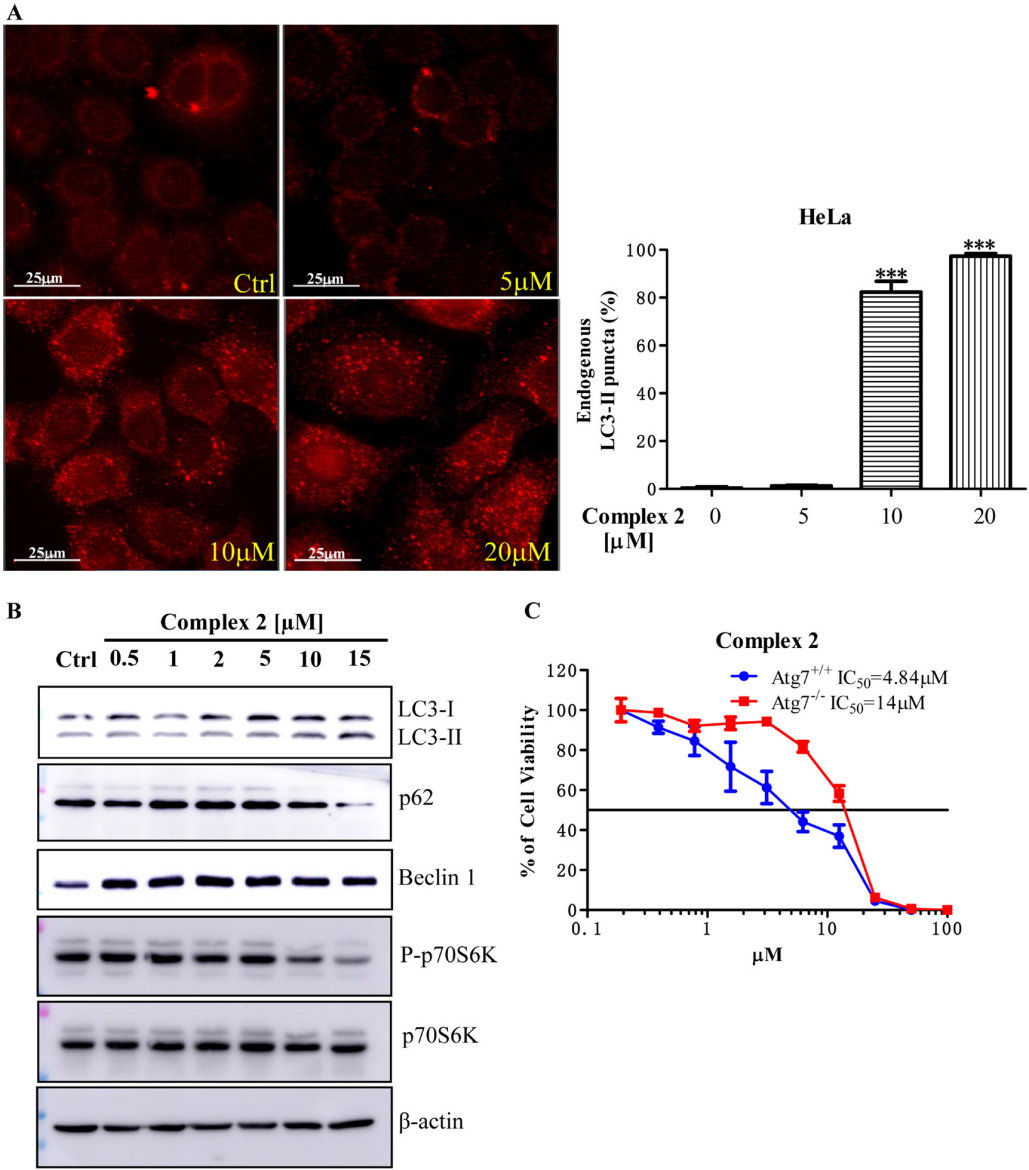
Cobalt complex 2 induces autophagy and autophagic cell death in cancer cells (**A**) Cobalt complex 2 does-dependently induced autophagy in HeLa cancer cells. HeLa cells treated with DMSO or indicated concentrations of cobalt complex 2 for 24 h were fixed and visualized for endogenous LC3-II expression by fluorescence microscopy using LC3-II antibody plus TRITC-conjugated anti-mouse secondary antibody. ***P < 0.001. Data were mean value ± S.D of three independent experiments. (**B**) Cobalt complex 2 induced LC3-II conversion in HeLa cancer cells. HeLa cells were treated with DMSO, or indicated concentrations of cobalt complex 2 for 24 h. Cell lysates were harvested and analyzed by Western blot for LC3 conversion (LC3-I, 18 kDa; LC3-II, 16 kDa) and β-actin. (**C**) Cytotoxicity of cobalt complex 2 in Atg7^+/+^ wild-type and Atg7^−/−^ deficient MEFs. Atg7^+/+^ wild-type and Atg7^−/−^ deficient MEFs were incubated with cobalt complex 2 for 72 h, MTT assay was performed to determine their cytotoxicity. The IC_50_ values shown on the chart are mean values of three independent experiments.

Autophagy is an evolutionarily conserved mechanism in which cellular proteins and organelles are eliminated through the lysosomal degradation pathway [28]. Deficiencies in autophagic pathway give rise to physiological dysfunction leading to DNA damage or chromatin instability, and eventually develop cancer [29]. Emerging evidence illustrated that autophagy acts as tumor suppressive mechanism, which induces autophagic cell death (type II programmed cell death) in cancer cells [30–33]. We therefore investigated whether the cobalt complexes could suppress cancer cells growth via autophagy induction. As shown in Figure 2A, the most potent cobalt complex 2 was found to induce autophagy in dose-dependent manner as indicated by increased percentages of red endogenous LC3-II puncta formation (red TRITC signal), whereas the cells without treatment (Ctrl) showed only slight or no red puncta formation. In addition, the protein conversion from LC3-I to LC3-II is the marker for autophagy induction. Here, we demonstrated that complex 2 dose-dependently increased the LC3-II protein conversion (Figure 2B). The expression of beclin-1 protein which is essential for autophagosome formation [34] was also upregulated after complex 2 treatments when compared to the untreated control (Figure 2B). On the other hand, p62 directly binds to LC3 and will be degraded by autophagy [35], together with the downstream target of mTOR, the phosphorylation of p70S6K (P-p70S6K), are other autophagy markers and the downregulated expression of the two markers imply autophagy activation [36, 37]. As illustrated in Figure 2B, addition of complex 2 to HeLa cells in the concentration of 10μM and 15μM prominently reduced the protein expression of both p62 and P-p70S6K. Such result is in line with the cellular immunofluorescence data observed in Figure 2A in which significant amount of red endogenous LC3-II puncta formation were induced in HeLa cells with complex 2 in concentration above 10 μM.

In order to further confirm the cobalt complex-induced autophagy is necessary for its anti-cancer effect, we therefore examined the cytotoxic effect of complex 2 in both autophagy-wild type and -deficient cells respectively. Therefore, both autophagy related gene (Atg) 7 wild-type and deficient mouse embryonic fibroblasts (MEFs) were used in this study [38]. Complex 2 was found to show much lower IC_50_ value in Atg7 wild-type MEFs (mean IC_50_, 4.84 μM) (Figure 2C), whereas less toxic effect in autophagy deficient cells (Atg7 deficient MEFs, mean IC_50_, 14 μM) was observed. In view of the fact that complex 2-mediated cytotoxicity was markedly abrogated from the failure of autophagy induction in Atg7^-/-^ deficient cells, these findings implicated that complex 2-induced autophagy would ultimately led to autophagic cell death.

**Figure 3:**
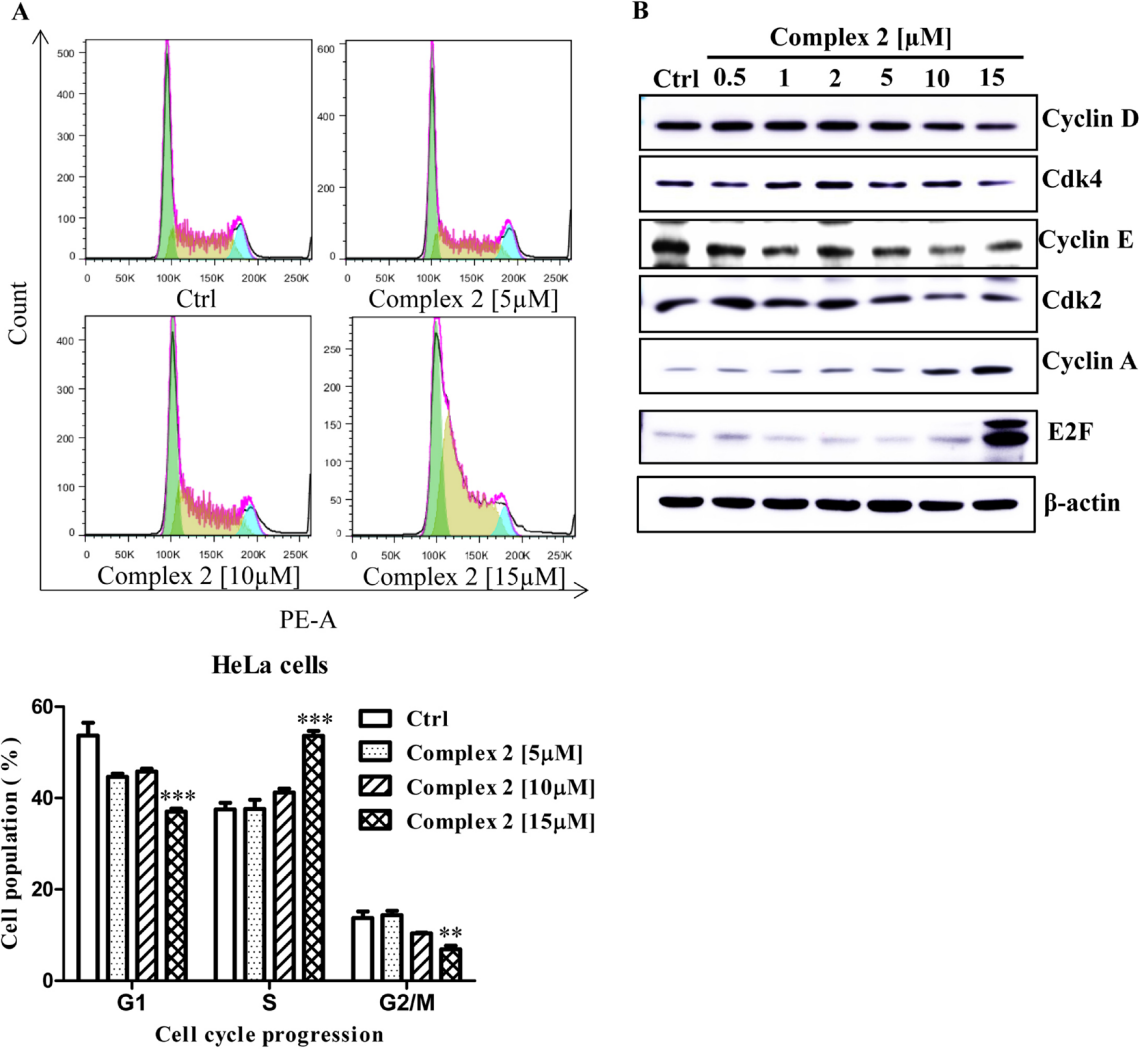
Cell cycle progression of cobalt complex 2 in HeLa cancer cells Exponentially growing HeLa cells were synchronized in the serum-free medium for 24 h. Then the cells were incubated with the DMSO and indicated concentrations of cobalt complex 2 for 24 h. (**A**) The cell cycle progression was evaluated using propidium iodide (PE-A) staining and flow cytometry analysis. The bar chart indicated the results of quantitative analysis of cell-cycle distribution (% of cell population). Means ± S.D. were from three independent experiments (One-way ANOVA: **P < 0.01, and ***P < 0.001). (**B**) Cobalt complex 2 altered the S phase specific cell cycle markers expression in HeLa cancer cells. HeLa cells were treated with DMSO or indicated concentrations of cobalt complex 2 for 24 h. Cell lysates were harvested and analyzed by Western blot for Cyclin D, Cyclin-dependent kinase 4 (Cdk4), Cyclin E, Cyclin-dependent kinase 2 (Cdk2), Cyclin A, E2F transcription factor, and β-actin.

To investigate whether the cobalt complex could suppress cancer cells growth by arresting cell cycle progression, HeLa cancer cells were treated with different concentrations of complex 2. Apparently, low concentration of complex 2 showed negligible effect on cell cycle progression. On the other hand, high concentration of complex 2 was found to arrest HeLa cancer cell in S-phase, as determined by the increased percentage of cell population accumulated in S-phase (Figure 3A), suggesting that this cobalt complex may affect the DNA replication process in cancer cells. To further demonstrate the effect of complex 2 in S-phase cell cycle arrest, the cytosolic level of cyclin D, Cdk4, cyclin E, Cdk2, cyclin A, and the E2F transcription factor were subjected to Western-blot analysis. These proteins are key regulators for controlling G1 to S phase cell cycle progression. During the course of G1 phase, the expression of cyclin D and E is upregulated, forming complex with Cdk4 and Cdk2, respectively, for supporting G1/S transition [39, 40]. Once the cell entered the S phase, cellular level of cyclin D and E, and Cdk4 decreased and is followed by the continuous expression of E2F-mediated cyclin A, which replace cyclin E to associate with Cdk2 for facilitating S phase progression into the G2 phase [41–43]. The expression of cyclin A eventually degraded when cell cycle successfully proceed to the G2 phase [41, 44]. Upon 24-hour exposures of our HeLa cell models to complex 2, at the concentration 10 and 15 μM, the protein level of cyclin D and E, cdk4, and cdk2 were decreased while significant accumulation of cyclin A and E2F were detected (Figure 3B). Such data suggested that complex 2 treatment intervene with the cell cycle machinery by stopping the cell from proliferation at the S phase.

**Figure 4:**
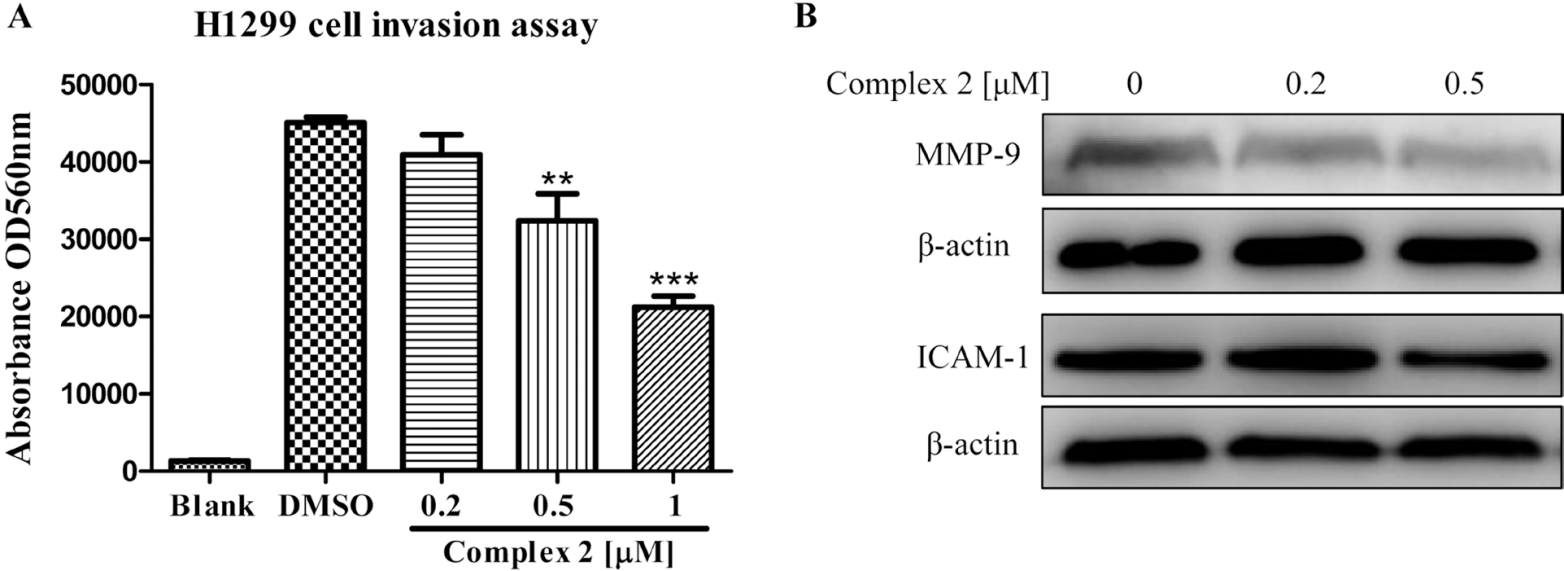
Cobalt complex 2 dose-dependently inhibits cancer cell invasion (**A**) H1299 lung cancer cells seeded in the ECMatrix^TM^ chamber were incubated with cobalt complex 2 for 72 h. The invaded H1299 cancer cells found in the lower layer of ECMatrixTM chamber were captured by digital camera under microscope with 40× magnifications. Bar chart represented the percentage of invasion ability of the stained invasive cells solute. Means ± S.D. were from three independent experiments. **P < 0.01 and ***P < 0.001 compared to DMSO control. (**B**) Cobalt complex 2 altered the expression of cell invasion markers in HeLa cancer cells. HeLa cells were treated with DMSO or indicated concentrations of cobalt complex 2 for 24 h. Cell lysates were harvested and analyzed by Western blot for Matrix metallopeptidase 9 (MMP-9), Intercellular Adhesion Molecule 1(ICAM-1) and β-actin.

To determine the inhibitory effect of cobalt complex in cancer cell invasion, human H1299 lung cancer cells with well-known cell invasion ability were treated with complex 2 in its sub-lethal (non-toxic) doses. Figure 4A shows the invasion assay of cancer cell H1299 upon the treatment of complex 2. The H1299 cancer cells treated with DMSO control demonstrated a significant number of invaded cells, as determined by the increased absorbance signal. It is noteworthy that complex 2 was found to dose-dependently decrease the absorbance signal. In addition, the expression profiles of matrix metalloproteinase-9 (MMP-9) and intercellular adhesion molecule-1 (ICAM-1) in complex 2-treated the H1299 cell were further analyzed by western-blot. MMP-9 and ICAM-1 are critical to the invasion and metastasis of cancers [45–47], therefore serve as markers for assessing the invasiveness of our cellular model. Consistent with the findings illustrated in Figure 4A, the application of complex 2 significantly downregulated the expression of both MMP-9 and ICAM-1 in H1299 cell at the concentration of 0.5 μM (Figure 4B). Taken together, our results suggested that cobalt complex 2 could suppress cancer cell invasion and metastasis.

### Cobalt complex 2 suppresses taxol-resistant cancers via P-gp inhibition

**Figure 5:**
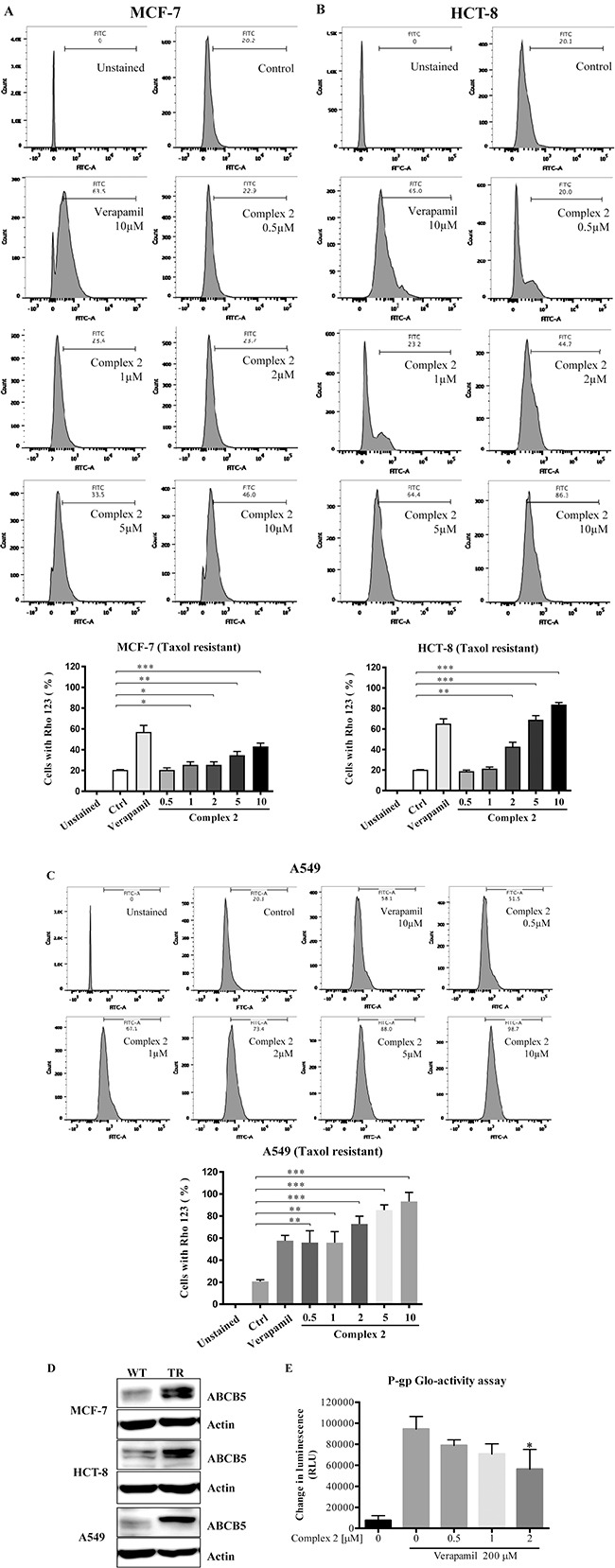
Cobalt complex 2 directly inhibits P-gp in Taxol-resistant cancer cells (**A**–**C**) Cobalt complex 2 inhibited P-gp activity in MCF-7, HCT-8, and A549 taxol-resistant cancer cells, respectively. Taxol-resistant cancer cells were incubated with DMSO, verapamil (10 μM), or indicated concentrations cobalt complex 2 for 4 h. The Rhodamine 123 (Rho-123) exclusion assay was determined using Rho-123 staining and flow cytometry analysis. Bar chart represented the quantitation of cells accumulated with Rho-123 dye. Means ± S.D. were from three independent experiments (One-way ANOVA: * *P* < 0.05, **P < 0.01, and ***P < 0.001). (**D**) MCF-7, HCT-8, and A549 taxol-resistant (TR) cancer cells indicated the over-expression of ATP-binding cassette member B5 (ABCB5). Cell lysates were harvested and analyzed by Western blot for ABCB5 and β-actin. (**E**) Cobalt Complex 2 directly inhibited P-gp *in vitro*. Cobalt Complex 2 (0–2 μM) were incubated with P-gp protein in the presence of a stimulatory drug, verapamil (200 μM), and ATP for 1 h at 37°C. The luminescence was then measured by Pgp-GloTM Assay System. Changes in luminescence (RLU) represented the P-gp ATPase activity. *P < 0.05 compared to verapamil alone.

The development of taxol-resistant phenotype of cancer cells is correlated to the degree of cellular P-gp expression associated with the upregulation of cytosolic drug efflux [48]. In order to examine the effect of complex 2 in P-gp activity in our taxol-resistant cancer cells, including MCF-7, HCT-8 and A549, Rhodamine 123 (Rho123) exclusion assay and flow cytometric analysis were used to investigate the accumulation of the Rho123 dye in these cells after complex 2 treatment. As shown in Figure 5A, 5B, and 5C, the accumulation of Rho123 dye could only be found in around 20% of the untreated taxol-resistant MCF-7, HCT-8 and A549 cells. In contrast, The P-gp activities of the three cell lines were inhibited and Rho123 accumulation were increased (up to c.a. 80%) after the addition of 10 μM of verapamil as positive control, indicating the involvement of P-gp pumps in the process. Complex 2 also induced inhibitory effects towards P-gp activity in all the treated cell lines and were in a dose-dependent manner as demonstrated by the increasing trend of Rho123 accumulation in these cellular models (Figure 5A, 5B, and 5C). To further validate our taxol-resistant models are in the multidrug-resistant phenotype, protein expression of the well-known cancerous multidrug resistance P-gp transporter, ATP binding cassette subfamily B member 5 (ABCB5) [49], was detected by Western blot analysis. All of the taxol-resistant cancer cells demonstrated overexpression of ABCB5 when compared with the taxol-sensitive counterparts (Figure 5D) suggested that the taxol-resistant MCF-7, HCT-8 and A549 are multidrug resistance, in part, due to ABCB5 overexpression. In addition, the mechanistic action of complex 2 was evaluated by the non-cell based P-gp Glo-activity assay to measure the ATP consumption profile of complex 2-treated P-gp protein. When P-gp is inhibited by the addition of complex 2, ATP consumption decreases which is represented as “change in luminescence” in our result. Therefore, the lower the consumption of ATP, the lower the “change in luminescence” will be observed which is correlated to inhibited P-gp ATPase activity. Verapamil were used to stimulate the P-gp ATPase activity prior to complex 2 treatments and we found that, complex 2 dose-dependently inhibited the ATPase activity of P-gp via direct interaction (Figure 5E).

### Tumor growth suppression in a mouse lung cancer xenograft model without observable adverse effects

**Figure 6:**
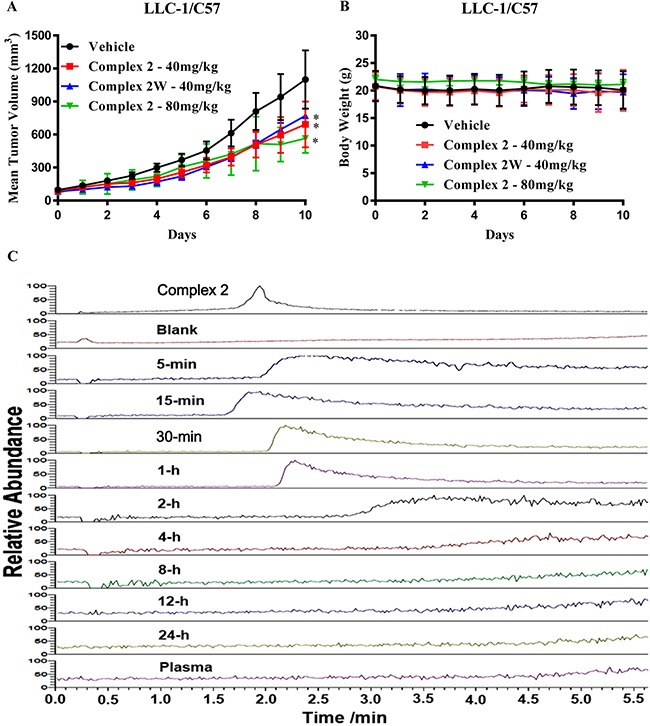
Cobalt complex 2 suppresses tumor growth in LLC-1 bearing mice (**A**) C57BL/6 mice were randomly grouped (*n* = 10) and intraperitoneal injected with 40 & 80 mg/kg of cobalt complex 2 or 40 mg/kg of water-soluble form of cobalt complex 2W, and vehicle everyday for 10 days, respectively. The comparison of the mean ± S.D. tumor volume was shown. Statistical differences were estimated using Student's *t*-test (two-tailed; two-sample equal variance). (**B**) Body weight of cobalt complex 2 or 2W-treated mice. (**C**) HPLC-MS profile of cobalt complex 2, blank, plasma and the blood samples with various time intervals. Monitored at the signal of m/z = 185.1071.

Complex 2 was further assessed in a lung cancer xenograft model in order to investigate its *in vivo* anti-tumor effect. As shown in Figure 6A, intraperitoneal (IP) injection of complex 2 or its water soluble form (complex 2W) at 40 mg/kg/day or at the high concentration at 80 mg/kg/day demonstrated significant tumor inhibitory effect of up to 50% (*P* < 0.05) of reduction in tumor volume. Treatment with complex 2 at both concentrations or 2W showed no significant reduction in body weight (Figure 6B), suggesting the less or non-toxic nature of the cobalt complex.

### Complex 2 is completely metabolized after absorption

The pharmacokinetic study of cobalt complex 2 in the mice was investigated. HPLC-MS was employed to analyze the chemical metabolism by monitoring the presence of cobalt complex 2 and/or its related chemical forms. The signals of m/z at 305.1604, 213.5607 and 185.1071, corresponding to [2]^2+^, [2 **–** Me_2_-bpy]^2+^ and [Me_2_-bpy]^+^, respectively, were monitored. In all blood samples, only the free ligand of Me_2_-bpy was detected while neither cobalt complex 2 nor its decomposed species was observed. The HPLC-MS profile was obtained accordingly by monitoring the signal of m/z at 185.1071, i.e. the free ligand signal (Figure 6C). The presence of such free ligand was attributed to the decomposition of cobalt complex 2 on the basis of the observation of same signal from the sample with additional cobalt complex 2. From the HPLC-MS profile, very weak or no detectable signal of the free ligand was detected in the samples after 2-hour time interval, suggesting of almost complete metabolism on this cobalt complex (Figure 6C). Therefore, the observed tumor inhibitory effects (Figure 6A) may be resulted from the decomposed species from complex 2.

## DISCUSSION

While the application of metal ions such as cisplatin in anti-cancer therapy has been fully studied in the 1960’s, further development of metal ions as anti-cancer therapeutic agents has also been reported [50, 51]. Combinational use of cisplatin has been applied to treat head and neck, cervical, ovarian, lung, and testicular cancers [52, 53]. Although platinum complexes can work as effective anti-cancer agents via the induction of tumor cell death, cisplatin might lead to toxicity of neural or renal cells, as well as bone marrow-suppression. Furthermore, various types of cisplatin resistance effects such as obstacle in cellular uptake, increased drug efflux, detoxification or DNA repair, and inhibition of apoptosis, were reported [21]. Other platinum analogs such as carboplatin and oxaliplatin, have been further developed and clinically approved with improved toxicity profile [54]. In light of this, studying the resistance mechanisms triggered by cisplatin is anticipated to explore more effective metal ions based drugs with reduced side effects. Jedlitschky *et al* reported that the multidrug resistance protein 2 (MRP2), which belongs to the ATP-binding cassette subfamily C, is participating in the efflux of cisplatin [55]. Similarly, cancer cell with arsenic-insensitive phenotype is associated with MRP2 and another multidrug transporter protein within the same family (MRP1) [56–61]. In contrast, our result demonstrated that cobalt complex 2 can interact and inhibit the activities of the P-gp protein. Such differences in cellular consequences induced by the various metallopharmaceuticals is mainly associated with the identity of the coordinating central metal [62] which suggested the clinical potential of cobalt ion-containnig complex in therapy of caners demonstrating multidrug resistance.

In fact, metal-ions based compounds have the abilities to coordinate ligands in a three dimensional configuration [1, 63], and provide suitable environment for various molecular structures that confer different geometries and kinetic properties that cannot be recognized by carbon-based chemical drugs [1, 64]. Malfunction of apoptosis is highly correlated to the development of drug resistance of cancer cells, therefore, targeting the anti-apoptotic proteins such as BCL 2 family members is important strategy of anti-cancer therapy. Although autophagy is responsible for maintaining normal cellular homeostasis during stressful conditions, excessive or uncontrolled regulation of autophagy can induce autophagy-dependent cell death, which is characterized by large-scale autophagic vacuolization of the cytoplasm [65].

In experimental mammalian cells settings, autophagy contributes to cell death with the absence of intact apoptosis machinery, for example, both etoposide and staurosporine can induce cell death in Bax^−/−^ Bak^−/−^ murine embryonic fibroblasts [66, 67]. Concomitantly, our previous findings have reported a group of natural alkaloids including liensinine, isoliensinine, cepharanthine and dauricine, which induced autophagy-dependent cell death in a panel of apoptosis-resistant cells [68]. Further evidence have confirmed novel natural compounds such as rottlerin, curcumin, genistein, quercetin, and resveratrol [69], STF-62247 [70] or guttiferone K [71], can regulate cancers through the autophagy dependent cell death [72, 73]. The regulatory role of resveratrol-induced autophagy in the crosstalk between apoptosis and cell cycle arrest, has further suggested the importance of tailor-making pharmacological interventions in cancer therapies [74]. Therefore, the induction of autophagy may work as an alternative way of inducing cell death in apoptosis-resistant cancer cells during cancer therapy. In this study, a panel of multidrug-resistant cancer cells from different origins was applied in order to confirm the anti-cancer properties of cobalt complexes towards multidrug-resistant cancers. With the indispensable role of metals in cellular regulation and pathogenesis of diseases, we have identified a panel of cobalt complexes that were able to specifically induce collateral sensitivity in taxol-resistant and p53-deficient cancer cells. Consistently, our reported anti-cancer functions of cobalt complexes 1–6 towards multidrug-resistant cancers have suggested the protective and non-toxic properties of cobalt metal-ions based compounds in anti-cancer therapies. As demonstrated in xenograft mouse model, our results also confirmed the identified cobalt complex 2 was able to suppress tumor growth *in vivo*. The anti-cancer effect of the cobalt complex was further demonstrated to be exerted via the induction of autophagy, cell cycle arrest and inhibition of cell invasion. Although limited researches were performed to study the role of metal ion in induction of autophagy, some findings suggested that autophagy is required for growth of fungus A. fumigatus under deficiency of metal ion, and proposed that autophagy is linked to homeostasis of metal ion [75]. Our data has therefore suggested innovative metal ion compounds for targeting drug resistance cancers in chemotherapies. The detailed mechanistic studies are in progress such as identification of drug targeting protein for further in-deep drug screening in order to explore the potential clinical application.

Collectively, the anti-cancer effect of cobalt complex 2 not only has been validated in different cancer and multidrug-resistant cancer models, but also in animal system. Our results therefore have provided the solid evidence to support the claims that the cobalt complexes would be developed into potential metal anti-cancer drug in the future.

## MATERIALS AND METHODS

### Reagents, chemicals, antibodies and plasmids

All chemicals and reagents were purchased from Sigma unless otherwise stated. The following regents were used: RIPA lysis buffer (CST, 9806), antibodies against LC3B (CST, 2775), anti-actin (Santa Cruz, sc-47778), ZyMax^TM^ TRITC-conjugated anti-mouse secondary antibodies (Invitrogen, PA1-28565). All solvents were of analytical reagent grade and purified according to standard procedures. NH_4_PF_6_ was purchased from Energy Chemical. NOBF_4_ was purchased from J&K Chemical.

### Synthesis and characterization

Complexes 3 and 4 were synthesized according to the reported method. Other cobalt(II) and cobalt(III) polypyridine complexes were prepared by the modification of a reported procedure [18].

[Co^II^(N^N)_3_](PF_6_)_2_. A mixture of CoCl_2_·6H_2_O (4 mmol) and 2,2’-bipyridine (13.2 mmol) in methanol (100 mL)were heated to reflux under N_2_ for 2 hours. After cooling down to room temperature, NH_4_PF_6_ (20 mmol) was added to the reaction mixture and the reaction mixture was stirred for another 1 hour. The precipitate was filtrated and washed with MeOH and then diethyl ether.

[Co^III^(N^N)_3_](PF_6_)_3_. Oxidation of [Co^II^(N^N)_3_](PF6)_2_ (2 mmol) was carried out by the treatment of NOBF_4_ (2 mmol) in acetonitrile (40 mL) at room temperature for 1 hour. After removing the solvent under reduced pressure, the residue was dissolved in acetonitrile (10 mL) and NH_4_PF_6_ (10 mmol) was added. The precipitate was collected by filtration and the solid was purified by recrystallization of acetone/Et_2_O to afford the crystal form of the desired product.

### [Co^II^(4,4’-Me_2_-bpy)_3_](PF_6_)_2_ (1)

Complex 1 has been obtained as yellow solid in 95% yield. Positive-ion ESI-MS ion cluster at *m/z* (%): 756.20 [M – PF_6_^‾^]^+^. IR (KBr) *v*/cm^-1^: 843 (PF_6_^‾^). Anal. Calcd for CoC_36_H_36_N_6_F_12_P_2_·H_2_O (%): C, 47.02; H, 4.17; N, 9.14. Found: C, 46.88; H, 4.14; N, 9.13.

### [Co^III^(4,4’-Me_2_-bpy)_3_](PF_6_)_3_ (2)

Complex 2 has been obtained as yellow crystals in 88% yield (through two steps). ^1^H NMR (300 MHz, DMSO-*d6*, 298K, TMS)/ppm: *δ* 8.91 (s, 2H, *H3* of bpy), 7.61 (d, 2H, *J* = 4.8 Hz, *H6* of bpy), 7.27 (d, 2H, *J* = 4.8 Hz, *H5* of bpy), 2.63 (s, 6H, *CH3*). ^13^C NMR (75 MHz, DMSO-*d6*, 298K, TMS)/ppm: *δ* 156.7, 155.2, 150.4, 132.2, 127.9, 21.5. IR (KBr) *v*/cm^-1^: 853 (PF_6_^‾^). Positive-ion ESI-MS ion cluster at *m/z*901.2 [M – PF_6_^‾^]^+^, 378.1 [M – 2 × PF_6_^‾^]^2+^, 203.7 [M – 3 × PF_6_^‾^]^3+^. Anal. Calcd for CoC_36_H_36_N_6_F_18_P_3_·MeOH(%): C, 41.20; H, 3.74; N, 7.79. Found: C, 41.09; H, 4.09; N, 7.82.

### [Co^III^(4,4’-(OMe)_2_-bpy)_3_](PF_6_)_3_ (5)

Complex **5** has been obtained as orange crystals in 87% yield(through two steps). ^1^H NMR (300 MHz, DMSO-*d6*, 298K, TMS)/ppm: *δ* 8.68 (s, 2H, *H3* of bpy), 7.32 (d, 2H, *J* = 5.1 Hz, *H6* of bpy), 7.21 (d, 2H, *J* = 5.1 Hz, *H5* of bpy), 4.09 (s, 6H, O*CH3*). ^13^C NMR (75 MHz, DMSO-*d6*, 298K, TMS)/ppm: *δ* 170.6, 156.8, 151.6, 117.4, 114.1, 58.2. IR (KBr) *v*/cm^–1^: 845 (PF_6_^‾^). Positive-ion ESI-MS ion cluster at *m/z* 997.1 [M – PF_6_^‾^]^+^, 426.1 [M – 2 × PF_6_^‾^]^+^, 235.7 [M – 3 × PF_6_^‾^]^+^. Anal. Calcd for CoC_36_H_36_O_6_N_6_F_18_P_3_·2H_2_O (%): C, 36.39; H, 3.42; N, 7.13. Found: C, 36.66; H, 3.50; N, 7.24.

### [Co^III^(4,4’-(C_9_H_19_)_2_-bpy)_3_](PF_6_)_3_ (6)

Complex **6** has been obtained as yellow crystals in 85% yield (through two steps). ^1^H NMR (300 MHz, DMSO-*d6*, 298K, TMS)/ppm: *δ* 8.95 (s, 2H, *H3* of bpy), 7.64 (d, 2H, *J* = 4.8 Hz, *H6* of bpy), 7.24 (d, 2H, *J* = 4.8 Hz, *H5* of bpy), 2.88 (t, 4H, *J* = 5.7 Hz, *CH2*), 1.71−1.67 (m, 4H, *CH2*), 1.31−1.20 (m, 24H, *CH2*), 0.87 (t, 6H, *J* = 5.1 Hz, *CH3*). ^13^C NMR (75 MHz, DMSO-*d6*, 298K, TMS)/ppm: *δ* 160.6, 155.5, 150.8, 131.3, 127.2, 35.0, 31.7, 29.7, 29.4, 29.2, 29.1, 22.6, 14.4.IR (KBr) *v*/cm^–1^: 835 (PF_6_^‾^). Positive-ion ESI-MS ion cluster at *m/z* 1574.9 [M – PF_6_^‾^]^+^, 714.5 [M – 2 × PF_6_^‾^]^2+^, 428.0 [M – 3 × PF_6_^‾^]^3+^. Anal. Calcd for CoC_84_H_132_N_6_F_18_P_3_·CH_3_CH_2_OCH_2_CH_3_ (%): C, 58.92; H, 7.98; N, 4.68. Found: C, 59.03; H, 7.93; N, 4.65.

### Physical measurements and instrumentation

^1^H NMR spectra were recorded on a Bruker AVANCE 400 Fourier-transform NMR spectrometer with chemical shifts reported relative to tetramethylsilane, (CH_3_)_4_Si. ESI mass spectra were performed on Orbitrap Fusion^TM^ Tribrid^TM^ Mass spectroscopy. Elemental analyses of the compounds were carried out on a Vario El cube elemental analyzer at the Sun Yat-Sen University Instrumental Analysis & Research Center, Guangdong, China.

### Cell culture

All cells were obtained from the American Type Culture Collection (Rockville, MD, USA) unless otherwise specified. Immortalized wild-type and Atg7-deficient mouse embryonic fibroblasts (MEFs) were kindly provided by Professor Masaaki Komatsu (Juntendo University, School of Medicine, Japan). Taxol-resistant types of MCF-7, HCT-8, A549 cancers cells and cisplatin-resistant SGC7901 cancer cells were purchased from KeyGEN BioTECH, China. Cisplatin-sensitive or –resistant A2780, OV2008 and C13 cancer cells were kindly provided by Prof. Benjamin Tsang (Department of Obstetrics and Gynecology and Department of Cellular and Molecular Medicine, University of Ottawa, Canada). HCT116 p53^+/+^ and p53^−/−^ isogenic human colon cancer cells were kindly provided by Professor Bert Vogelstein (Ludwig Center at Johns Hopkins, Howard Hughes Medical Institute, USA). All media were supplemented with 10% fetal bovine serum and antibiotics penicillin (50 U/ml) and streptomycin (50 μg/ml; Invitrogen, Paisley, Scotland, UK). All cell cultures were incubated at 37°C in a 5% humidified CO_2_ incubator.

### Cytotoxicity assays

All tested compounds were dissolved in DMSO at a final concentration of 50 mmol/L and stored at −20°C before use. Cytotoxicity was assessed by using the 3-(4,5-dimethylthiazol-2-yl)-2, 5-diphenyltetrazolium bromide (MTT) (5.0 mg/ml) assay as previously described [38]. Briefly, 4×10^3^ cells per well were seeded in 96-well plates before drug treatments. After overnight culture, the cells were then exposed to different concentrations of selected compounds (0.039–100 mmol/L) for 72 hours. Cells without drug treatment were used as control. Subsequently, MTT (10 μL) solution was added to each well and incubated at 37°C for 4 hours followed by the addition of 100 μL solubilization buffer (10%SDS in 0.01 mol/L HCl) and overnight incubation. A_570_ nm was then determined in each well on the next day. The percentage of cell viability was calculated using the following formula: Cell viability (%) = A_treated_/A_control_ × 100. Data were obtained from three independent experiments.

### Endogenous autophagy detection

The detection of endogenous LC3 was conducted using immunofluorescence staining method as described below. In brief, cobalt complex 2-treated HeLa cancer cells on cover slips were fixed with 4% paraformaldehyde (Sigma) for 20 min at room temperature and then rinsed with PBS. Coverslips were immersed in methanol at room temperature for 2 min. After washing with PBS, the cells were then incubated with anti-LC3 (1:200) in TBST (100 mM Tris HCl, pH 7.5, 150 mM NaCl, 0.05% Tween 20 and 5% BSA) overnight at 4^°^C. After washing with PBS, the cells were incubated with anti-mouse secondary antibody (TRITC) (1:200) in TBST containing 5% BSA at 37°C for 1 hour in the dark. The coverslips were then mounted with FluorSave™ mounting media (Calbiochem, San Diego, CA, USA) for fluorescence imaging and localization of LC3 autophagosomes were captured under the API Delta Vision Live-cell Imaging System (Applied Precision Inc.,GE Healthcare Company, Washington, USA). To quantify autophagy, guidelines were followed [76]. The percentage of cells with autophagic induction was calculated by the number of the cells with increased formation of punctate LC3 fluorescence dots (≥ 10 dots/cell) over the total number of immunofluorescence-positive cells in the same field. A minimum of 1000 cells from randomly selected fields were scored.

### Immunoblot analysis

Western blot analysis was carried out following standard methods. Cells were lysed with RIPA lysis buffer with protease and phosphatase inhibitor cocktails. Protein concentrations were determined using the Bio-Rad protein assay (Bio-Rad Laboratories, Inc., Hercules, CA, USA). After electrophoresis, the proteins from SDS/PAGE were electro-transferred to a Hybond enhanced chemiluminescence nitrocellulose membrane (Amersham Biosciences, NJ, USA), which was then blocked with 5% dried milk for 1 hour. After washing, the blot was incubated with the indicated primary antibodies overnight at 4°C. Detection was performed using appropriated HRP-conjugated secondary antibodies for 1 hour at RT followed by chemiluminescence (Invitrogen).

### Flow cytometry cell-cycle analysis

Cobalt complex 2-treated cells were harvested and analysed by flow cytometry using propidium iodide staining (BD Biosciences, San Jose, CA, USA) according to the manufacturer instructions. Cell population in different phase of cell cycle were quantitatively counted by a flow cytometer (BD FACSAria III, San Jose, CA, USA). Data acquisition and analysis were performed with CellQuest (BD Biosciences, San Jose, CA, USA) from triple independent experiments.

### Cell invasion assay

The H1299 cancer cell invasion assay was performed in a Cell Invasion Chamber, a 24-well tissue culture plate with cell culture inserts that contain an 8 μm pore size polycarbonate membrane over a thin layer of dried ECMatrix^TM^ (CHEMICON). H1299 cells (15000 cells/well) were re-suspended in serum-free medium and incubated in an invasion chamber insert with different concentrations of cobalt complex 2 for 72 hours, while the lower chamber contained medium with 10% FBS. The cells invaded through the ECM layer to the bottom of the polycarbonate membrane were labelled with Cell Stain provided in the kit for 20 min at room temperature. The non-invading cells were gently removed from the interior of the inserts by using a cotton-tipped swab. The number of invaded cells was counted through the microscope and quantified by dissolving stained cells in 10% acetic acid (200 μL/well). The colorimetric reading of the solute mixture was determined by spectrophotometer at OD 560 nm.

### Rhodamine 123 exclusion assay

Taxol-resistant cancer cells were seeded in 6 well plate with 2 × 10^5^ cells each well and cultured for 24 h at 37°C in an atmosphere containing 5% CO_2_. At confluence, taxol-resistant cancer cells were incubated with or without the modulator (10 μM of verapamil) and drug (0.5, 1, 2, 5 and 10 μM of cobalt complex 2) for 4 h at 37°C. Subsequently, 5 μg/mL of Rho123 was added to each well and the wells were incubated for another 1 h at 37°C. The accumulation of Rho123 was stopped by washing the cells five times with ice-cold PBS. The cells were then resuspended in 400 μL PBS for flow cytometry analysis. Intracellular fluorescence was measured using a flow cytometer at an excitation wavelength of 488 nm and emission wavelength of 525 nm. All data acquisition and analysis were performed with CellQuest (BD Biosciences, San Jose, CA, USA) with at least three independent experiments. Results were shown as the mean of fluorescence intensity.

### P-gp ATPase assay

Activity of P-gp ATPase in response to cobalt complex 2 or verapamil was determined by Pgp-Glo assay system (Promega, Madison, WI). According to the manufacture instruction, the inhibitory effect of cobalt complex 2 on the activity of P-gp ATPase was measured in the presence of verapamil (as a positive stimulator). The luminescence of the sample reflects the ATP level in the sample, which is negatively correlated with the activity of P-gp ATPase and was recorded using the SpectraMax Paradigm Multi-Mode Microplate Reader (Molecular Devices). DMSO-treated activities are expressed as the percentage of basal activity. By comparing basal activity to test compound-treated activities, the compounds can be ranked as stimulating, inhibiting, or having no effect on basal P-gp ATPase activity.

### LLC-1 xenograft tumor model

The mice were maintained under specific pathogen-free condition in a 12-h light-dark cycle. 8-week-old C57BL/6 mice (female body weight: 22 ± 4 g and male body weight 25 ± 4g) were purchased from The Chinese University of Hong Kong. The animals were housed in a 12 hours light/dark cycles and temperature-controlled room and given ad libitum access to food and water. All of the experiments were carried out in accordance to the “Institutional Animal Care and User Committee guidelines” of the Macau University of Science and Technology. C57BL/6 were randomly grouped (10 mice per group) and intraperitoneal injected with 40 mg/kg or 80 mg/kg of cobalt complex 2 or 40 mg/kg or 80 mg/kg of water-soluble form of cobalt complex 2. Xenograft mouse model was constructed as previously described [77]. LLC-1 Lewis lung cancer cells (1 × 10^6^ cells) were subcutaneously injected in the right dorsal region of the mice. After tumors grew to about 100 mm^3^, the mice were randomly divided into 3 groups: 1) the control group, 2) 40 or 80 mg/kg cobalt complex 2 group, and 3) 40 mg/kg cobalt complex 2W (water soluble form). Cobalt complex 2 was dissolved in 100 μL buffer (PEG400:EtOH:ddH_2_O = 6:1:3), whereas 2W (water soluble form) was dissolved in ddH_2_O. The complexes were then intraperitoneal injected in mice every day. The body weight of each mouse was recorded with the tumor volume determined by Vernier caliper using the formula of L × W^2^ × 0.52, where Lis the longest diameter of tumor and W is the shortest diameter of tumor. Mice with tumor implants were sacrificed after 10 days when the tumor size of vehicle control group reached above 1000 mm^3^ due to animal ethical issue.

### Pharmacokinetic study

Pharmacokinetic study of cobalt complex 2 was performed in male C57BL/6 mice by the dosage of 40 mg/kg. The source of mouse and practice of husbandry were the same as described in the methodology section of LLC-1 Xenograft Tumor experiment. Cobalt complex 2 was diluted in the formula (PEG400: H_2_O: Ethanol = 6:3:1) and administered by intraperitoneal in a volume of 0.005 mL/g bodyweight. After dosing, blood samples were collected to sodium heparin anticoagulant tube at different time points (5, 15, 30 min, and then 1, 2, 4, 8, 12 and 24 hours); 4 mice were used for each time point. Approximately 200 μL of blood was collected via orbital vein from each anesthetized mouse by isoflurane. Plasma was separated by centrifugation at 3000 g for 10 minutes at 4°C. The plasma was protected and stored at −80°C until analyzed by liquid chromatography-mass spectrometry/mass spectrometry (LC-MS/MS) for quantification.

Plasma samples were thawed at room temperature and then vortexed to ensure sample solution homogeneity. Firstly, a 20 μL aliquot of each plasma sample were loaded into 1.5 mL tube, 100 μL acetonitrile was added, vortexed then centrifuged at 13000 rpm for 10 min. the supernatant was collected. The plasma cobalt complex 2 concentrations were quantified using Thermo HPLC Ultimate 3000 system. The compound in plasma samples were analyzed by using the Agilent Zorbax Eclipse Plus C-18 column with a particle size of 1.8 μm. The parameters of the gradient elution program were applied as follows: mobile phase A (0.1% formic acid in water) and mobile phase B acetonitrile: 0–5.5 min, 20% B; 5.5–7 min, 90% B; 7–10 min, 20% B. The column and autosampler temperature were maintained at 40 and 4^°^C, respectively. The gas temperature was set 320^°^C with flow rate (0.8 mL/min), gases were set at 10 psi for the nebulizer, capillary, 3500 V.

### Statistical analysis

The results were expressed as means ± S.D. as indicated. The difference was considered statistically significant when the *p*-value was less than 0.05. Student's *t*-test or one-way ANOVA analysis was used for comparison among different groups.

## SUPPLEMENTARY MATERIALS FIGURES



## Abbreviations

MDR: Multidrug resistance
P-gp: P-glycoprotein
Atg: Autophagy related gene
MEFs: Mouse embryonic fibroblasts
MMP-9: Matrix metalloproteinase-9
ICAM-1: Intracellular adhesion molecule-1
Rho 123: Rhodamine 123
ABCB5: ATP binding cassette subfamily B member 5
IP: Intraperitoneal
MRP2: Multidrug resistance protein 2.
