# Supplementary material for: New perspectives of cobalt tris(bipyridine) system: anti-cancer effect and its collateral sensitivity towards multidrug-resistant (MDR) cancers
Source: Oncotarget. 2017 Jul 5;8(33):55003–21. doi: 10.18632/oncotarget.18991 (PMC5589637; doi:10.18632/oncotarget.18991)
Supplement: Supplementary file 1 [file oncotarget-08-55003-s001.pdf]

New perspectives of cobalt tris(bipyridine) system: anti-cancer effect and its collateral sensitivity towards multidrug-resistant (MDR) cancers

SUPPLEMENTARY MATERIALS

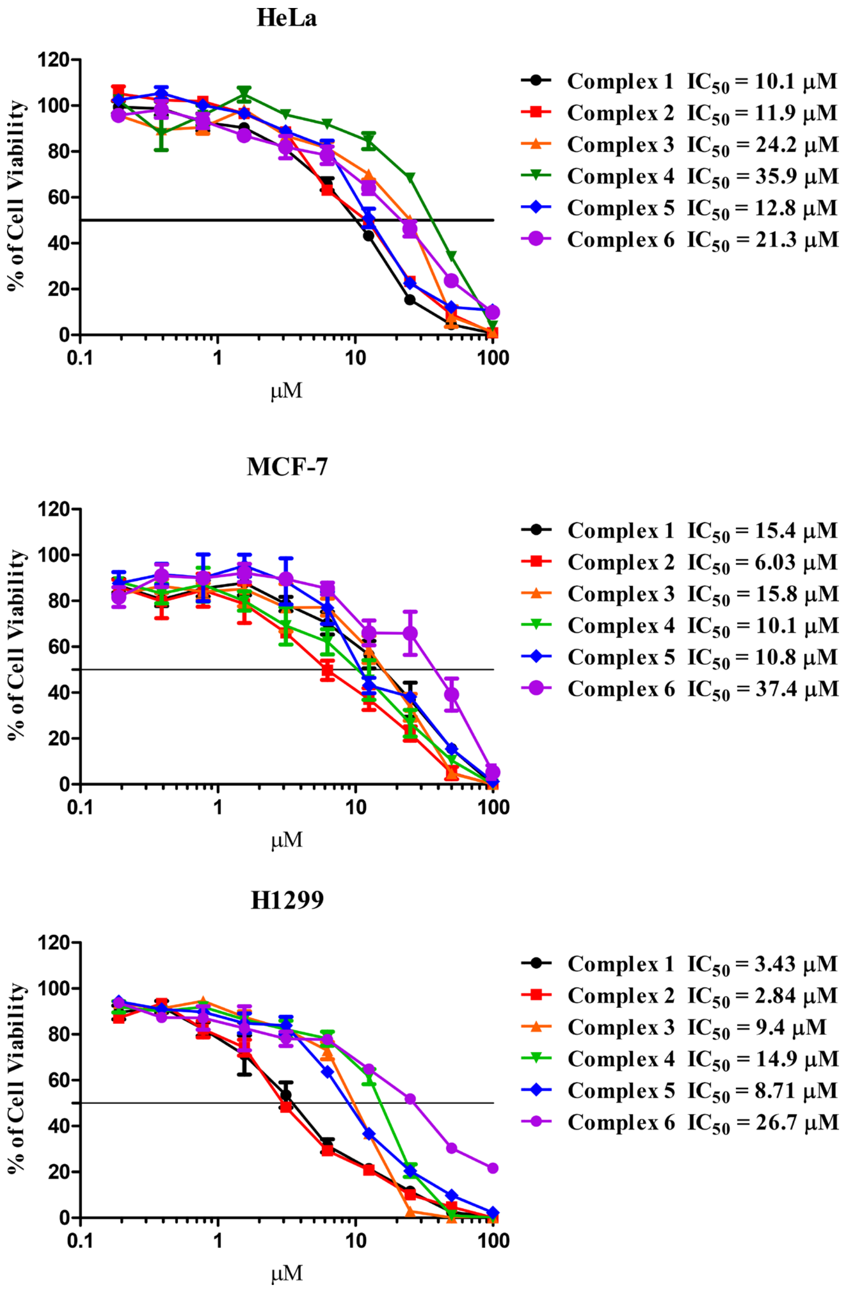

Supplementary Figure 1: Table 1 cytotoxicity of cobalt complexes 1–6 in HeLa, MCF-7, and H1299 cell lines. Cells were incubated with cobalt complexes 1–6 for 72 h, MTT assay was performed to determine their cytotoxicity. The IC_{50} values shown on the chart are mean values of three independent experiments.

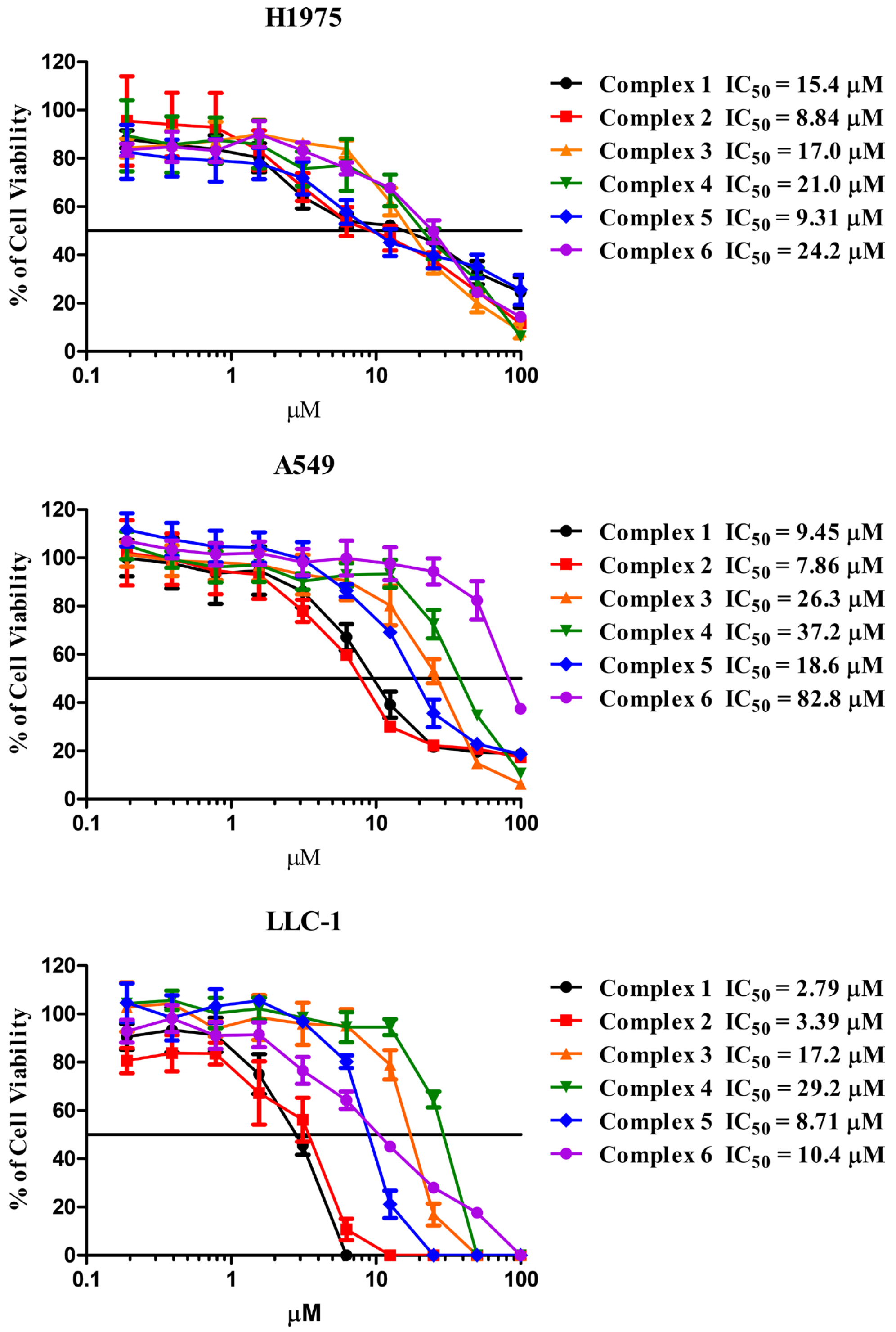

Supplementary Figure 2: Table 1 cytotoxicity of cobalt complexes 1–6 in H1975, A549, and LLC-1 cell lines. Cells were incubated with cobalt complexes 1–6 for 72 h, MTT assay was performed to determine their cytotoxicity. The IC_{50} values shown on the chart are mean values of three independent experiments.

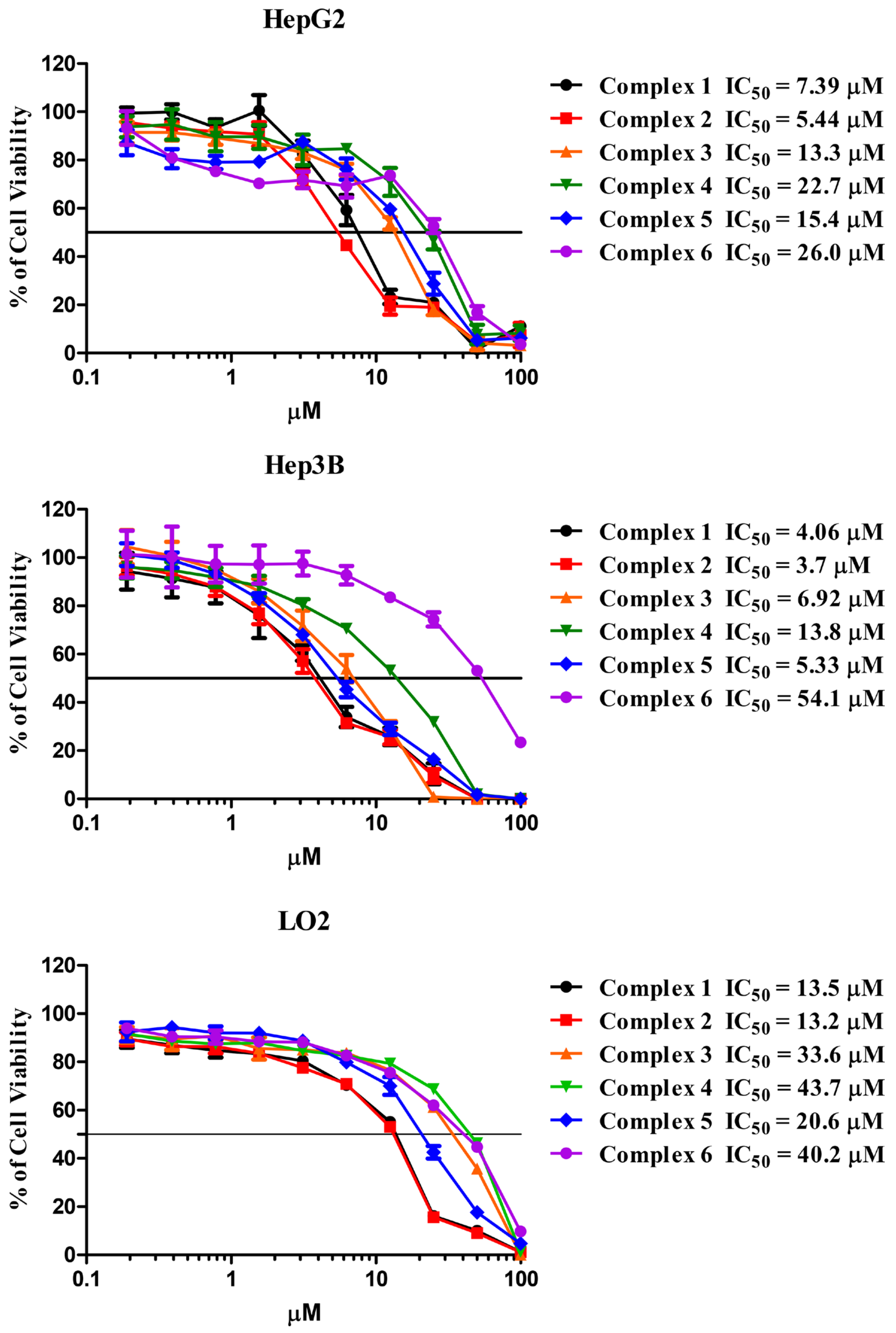

Supplementary Figure 3: Table 1 cytotoxicity of cobalt complexes 1–6 in HepG2, Hep 3B, and LO2 cell lines. Cells were incubated with cobalt complexes 1–6 for 72 h, MTT assay was performed to determine their cytotoxicity. The IC_{50} values shown on the chart are mean values of three independent experiments.

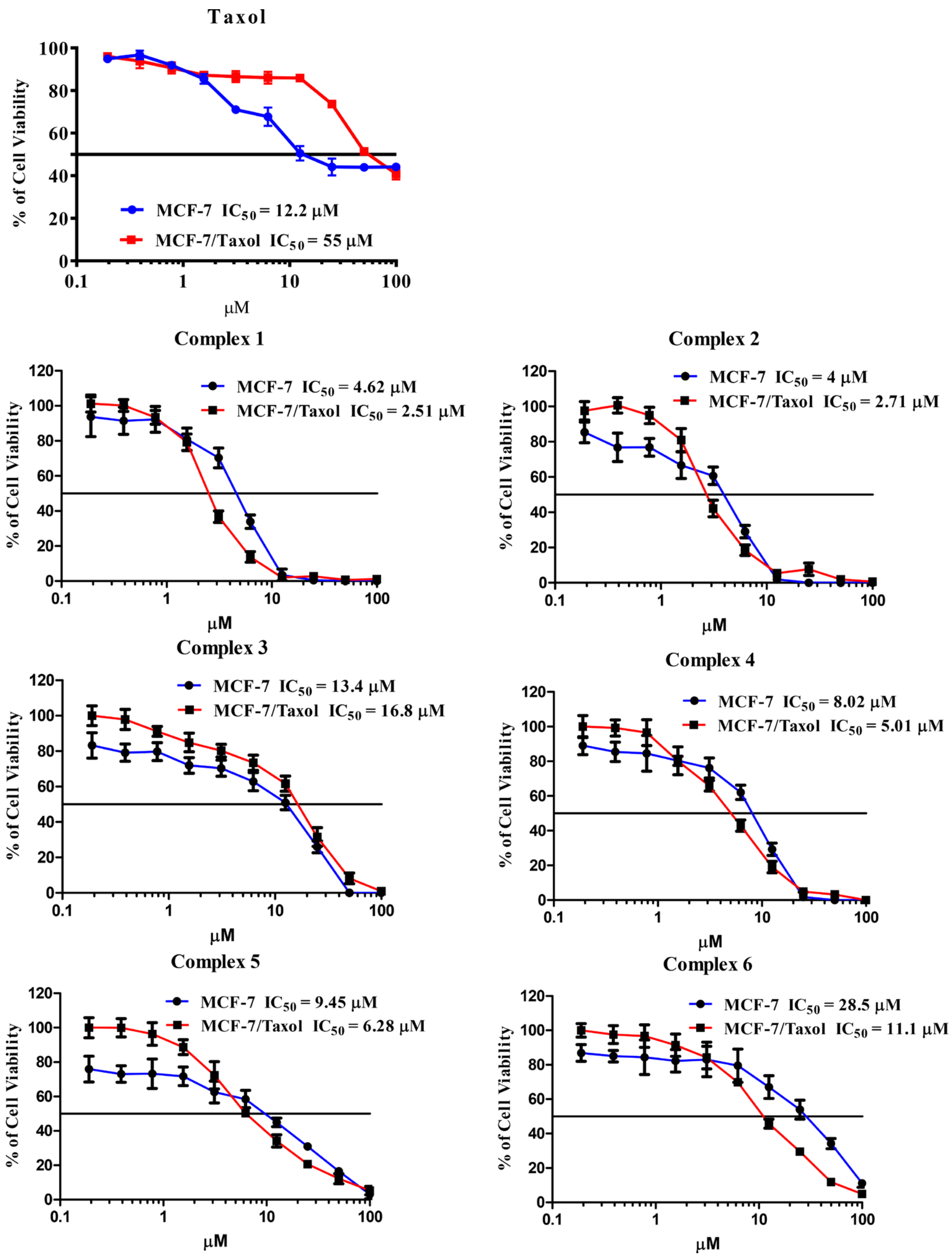

Supplementary Figure 4: Table 2 cytotoxicity of cobalt complexes 1–6 or taxol in MCF-7 wild type and taxol resistant cell lines. Cells were incubated with cobalt complexes 1–6 or taxol for 72 h, MTT assay was performed to determine their cytotoxicity. The IC_{50} values shown on the chart are mean values of three independent experiments.

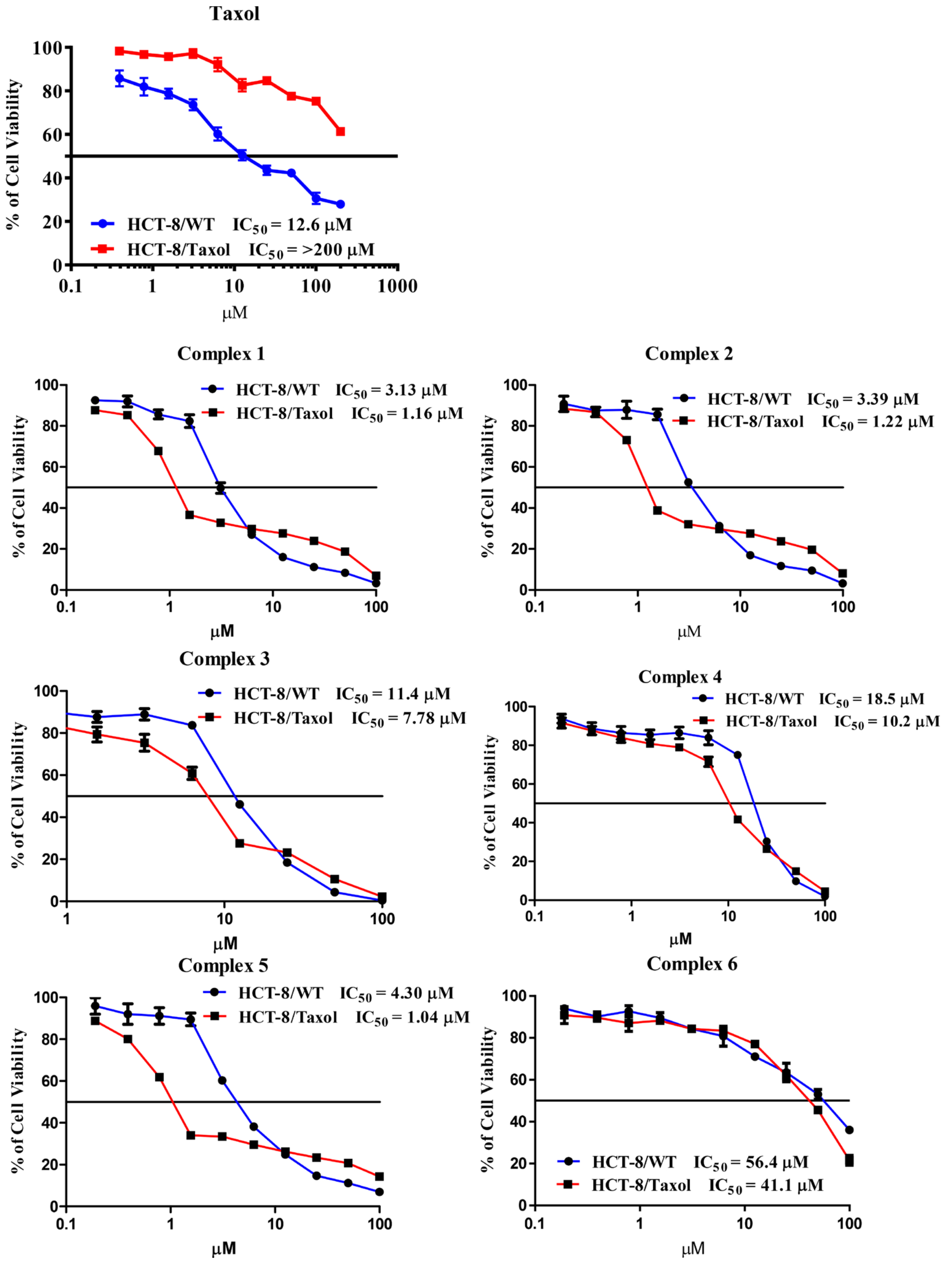

Supplementary Figure 5: Table 2 cytotoxicity of cobalt complexes 1–6 or taxol in HCT-8 wild type and taxol resistant cell lines. Cells were incubated with cobalt complexes 1–6 or taxol for 72 h, MTT assay was performed to determine their cytotoxicity. The IC_{50} values shown on the chart are mean values of three independent experiments.

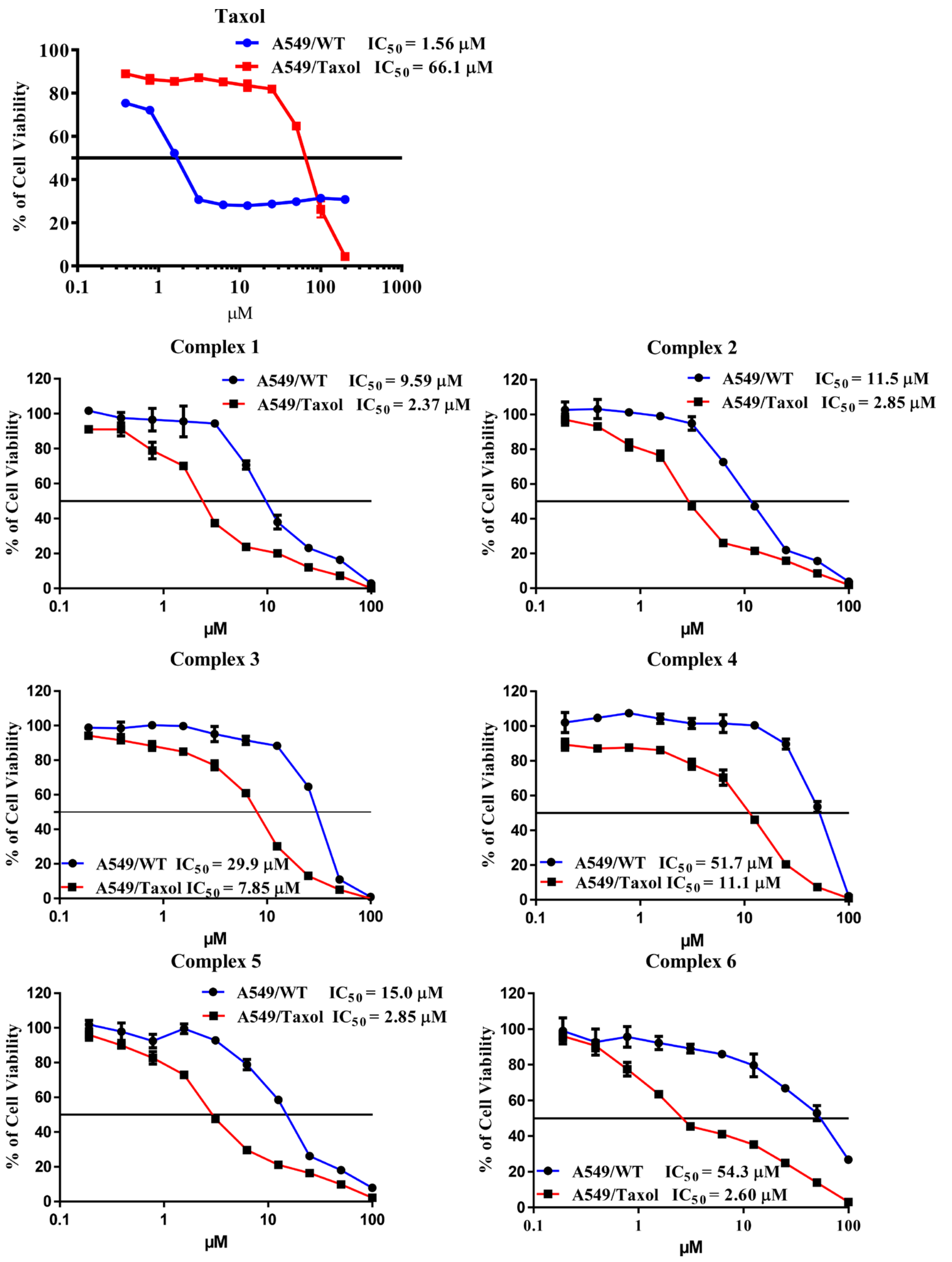

Supplementary Figure 6: Table 2 cytotoxicity of cobalt complexes 1–6 or taxol in A549 wild type and taxol resistant cell lines. Cells were incubated with cobalt complexes 1–6 or taxol for 72 h, MTT assay was performed to determine their cytotoxicity. The IC_{50} values shown on the chart are mean values of three independent experiments.

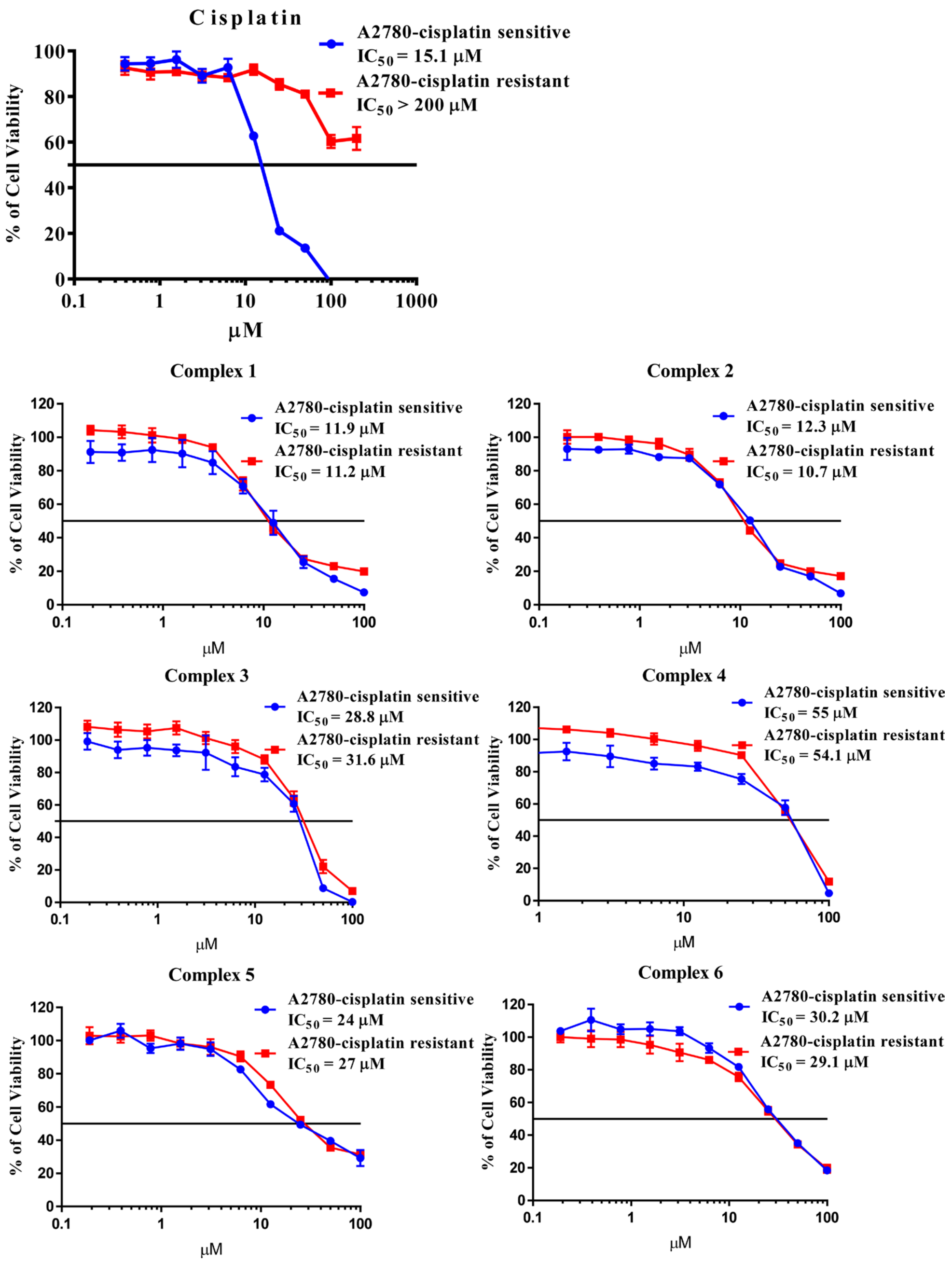

Supplementary Figure 7: Table 3 cytotoxicity of cobalt complexes 1–6 or cisplatin in A2780 sensitive and cisplatin resistant cell lines. Cells were incubated with cobalt complexes 1–6 or cisplatin for 72 h, MTT assay was performed to determine their cytotoxicity. The IC_{50} values shown on the chart are mean values of three independent experiments.

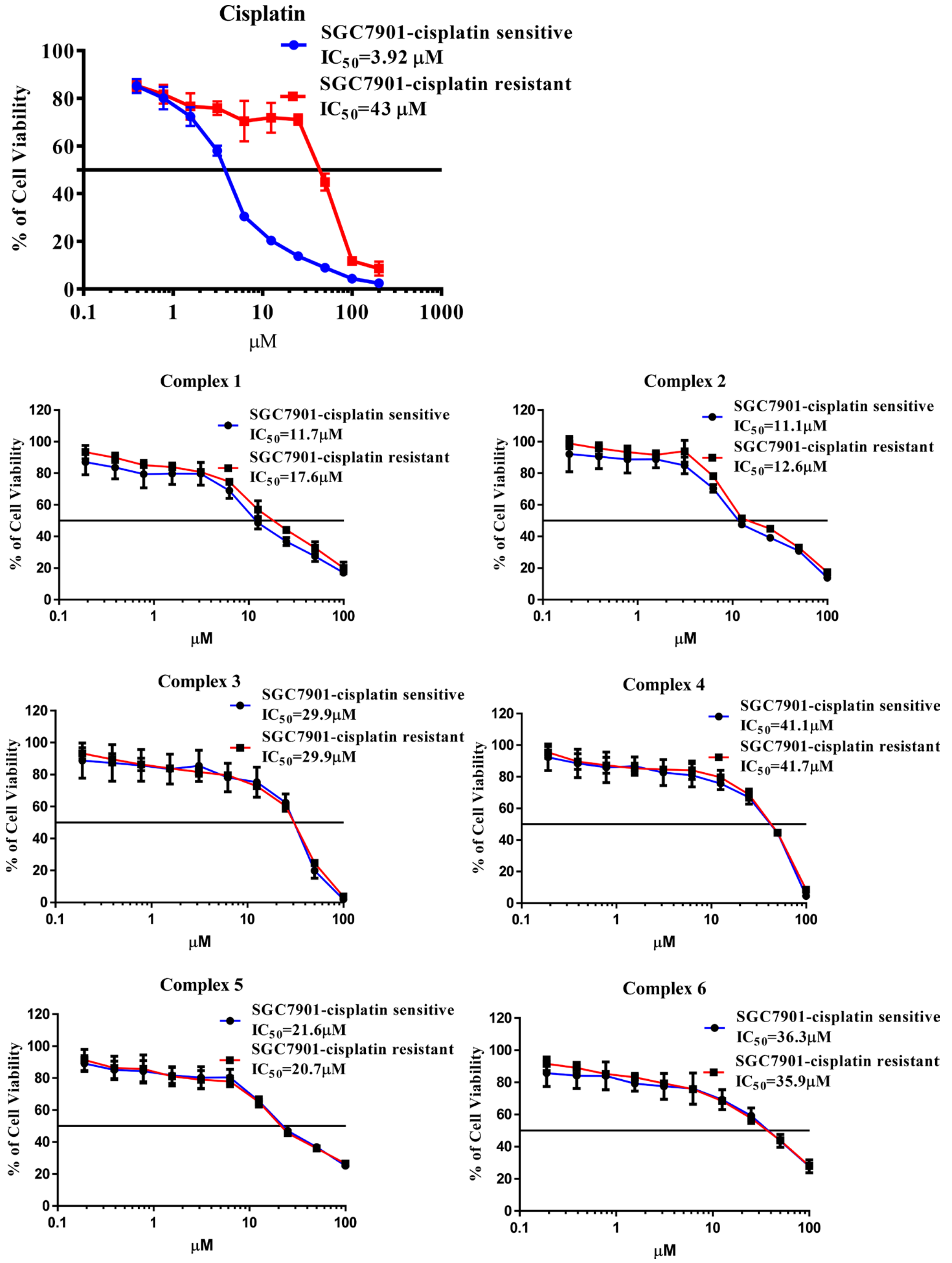

Supplementary Figure 8: Table 3 cytotoxicity of cobalt complexes 1–6 or cisplatin in SGC-7901 sensitive and cisplatin resistant cell lines. Cells were incubated with cobalt complexes 1–6 or cisplatin for 72 h, MTT assay was performed to determine their cytotoxicity. The IC_{50} values shown on the chart are mean values of three independent experiments.

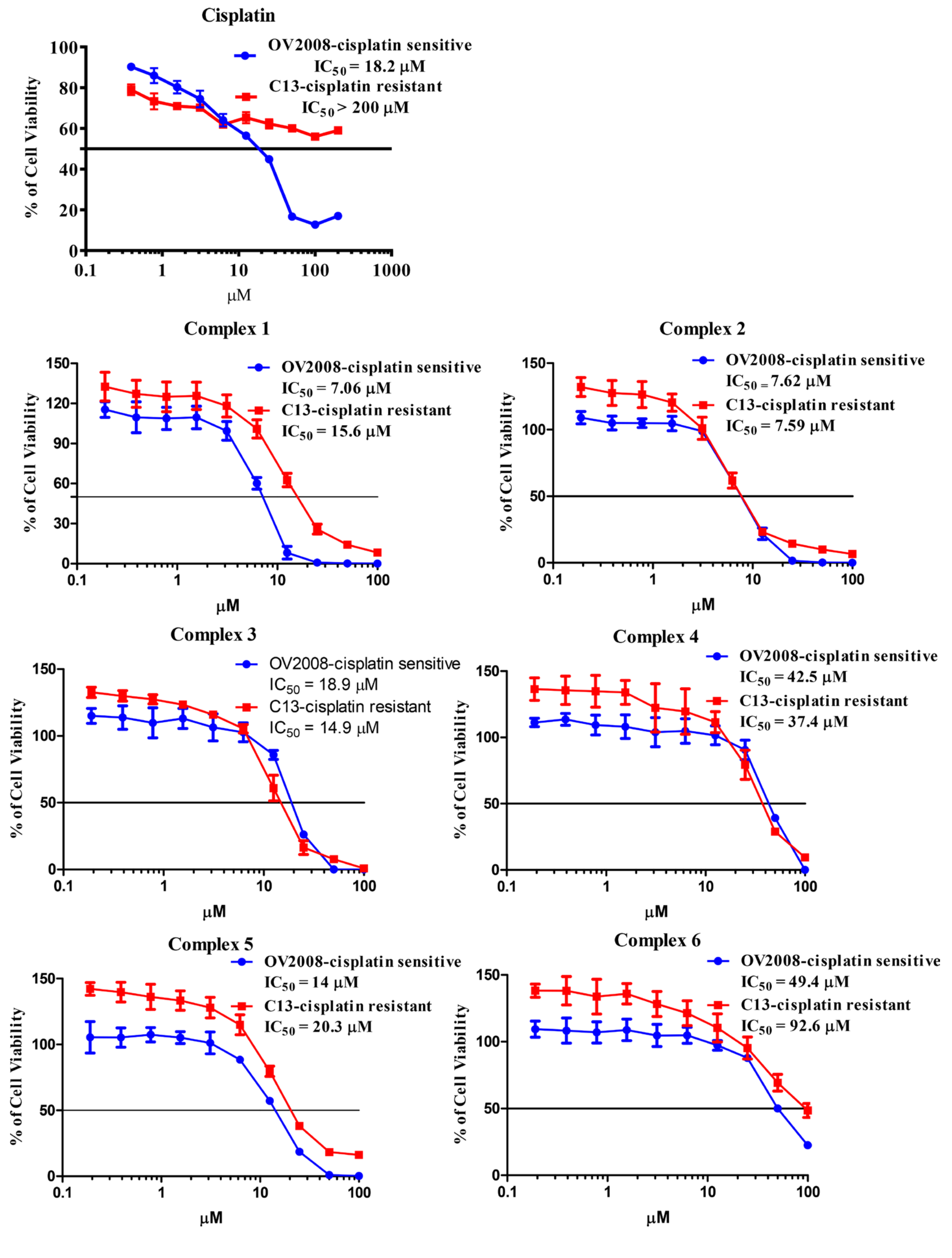

Supplementary Figure 9: Table 3 cytotoxicity of cobalt complexes 1–6 or cisplatin in OV2008 (cisplatin-sensitive) and C13 (cisplatin-resistant) cell lines. Cells were incubated with cobalt complexes 1–6 or cisplatin for 72 h, MTT assay was performed to determine their cytotoxicity. The IC_{50} values shown on the chart are mean values of three independent experiments.

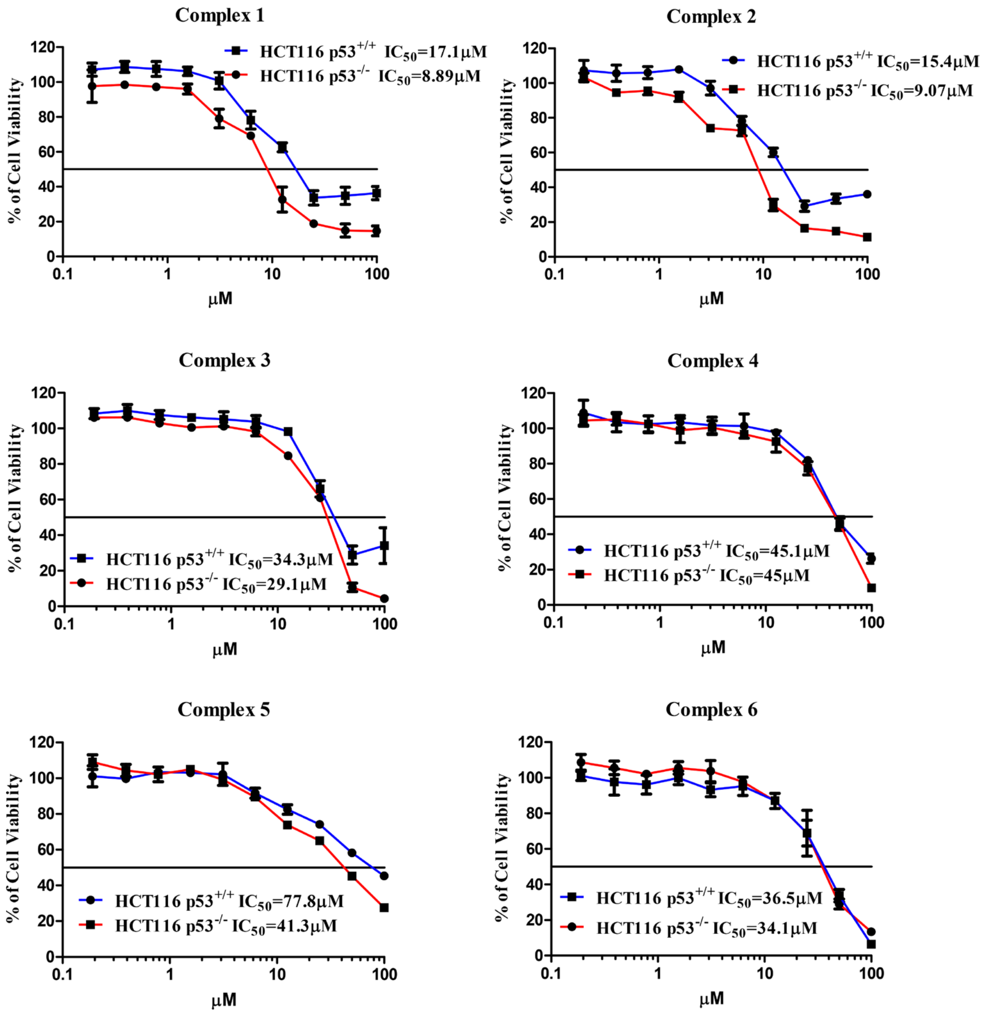

Supplementary Figure 10: Table 4 cytotoxicity of cobalt complexes 1–6 in HCT116 $p53^{+/+}$ and HCT116 $p53^{-/-}$ colon cancer cell lines. Cells were incubated with cobalt complexes 1–6 for 72 h, MTT assay was performed to determine their cytotoxicity. The IC_{50} values shown on the chart are mean values of three independent experiments.
